# *In Vivo* Multicellular Feedback Control in Synthetic Microbial Consortia

**DOI:** 10.1021/acssynbio.4c00862

**Published:** 2025-06-11

**Authors:** Davide Salzano, Barbara Shannon, Claire Grierson, Lucia Marucci, Nigel J. Savery, Mario di Bernardo

**Affiliations:** † Scuola Superiore Meridionale, 80134 Naples, Italy; ‡ School of Biochemistry, 1980 University of Bristol, Bristol BS8 1TD, United Kingdom; § School of Biological Sciences, University of Bristol, Bristol BS8 1TQ, United Kingdom; ∥ Bristol BioDesign Institute, University of Bristol, Bristol BS8 1QU, United Kingdom; ⊥ School of Engineering Mathematics and Technology, University of Bristol, Bristol BS8 1TW, United Kingdom; # School of Cellular and Molecular Medicine, University of Bristol, Bristol BS8 1TD, United Kingdom; ∇ Department of Electrical Engineering and Information Technology, 9307 University of Naples Federico II, Napoli 80125, Italy

**Keywords:** cybergenetics, synthetic biology, control engineering, multicellular control, synthetic microbial consortia, gene regulation, Escherichia coli, modularity

## Abstract

In this paper, we present a biomolecular control architecture able to guarantee stable and precise regulation of gene expression. Specifically, we engineer a microbial consortium comprising a cellular population, named *controllers*, that is tasked to regulate the expression of a gene in a second population, termed *targets*. Traditional biomolecular control strategies, while effective, are predominantly confined to single-cell applications, limiting their complexity and adaptability due to factors such as competition for limited cell resources and incompatible chemical reactions. Our approach overcomes these limitations by employing a distributed multicellular feedback loop between two strains of , allowing for division of labor across the consortium. *In vivo* experiments demonstrate that this control system maintains precise and robust gene expression in the target population, even amid variations in consortium composition. Our study fills a critical gap in synthetic biology and paves the way for more complex and reliable applications in the field.

## Graphical abstract

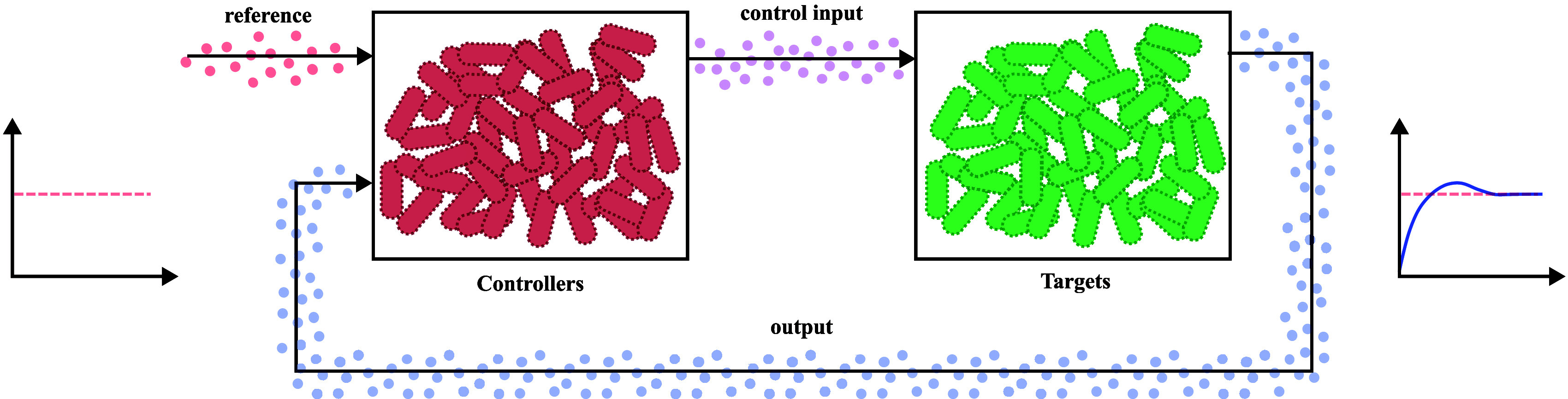

## Introduction

Synthetic biology aims to engineer biomolecular systems that equip cells with novel functions,^1^ offering potential applications across various domains. These range from designing bacteria capable of producing biofuels or sensing and degrading pollutants in the environment (such as hydrocarbons and plastics), to engineering immune cells that can track and kill cancer cells, or that can release drugs at specific points in desired conditions to avoid side effects (see ref 2 for a comprehensive review). A fundamental mechanism investigated in system and synthetic biology is feedback, which underpins many natural processes (*e.g.*, homeostasis in the human body). Feedback has been extensively harnessed to realize reliable and robust synthetic biology devices, increasing their flexibility and modularity.^3–5^

Control strategies for synthetic biological systems are typically implemented either internally (embedded or multicellular controllers) or externally (external controllers). External control systems rely on algorithms running on computers, which interface with cellular populations through an experimental platform that monitors the cells’ state and modifies their phenotype by applying external stimuli in real time. This approach provides excellent static and dynamic performance in closed-loop.^6^ However, it requires precise and continuous control of the cells’ growth environment, which may not be feasible for certain applications (see ref 5 and references therein). Moreover, as highlighted in ref 4, external controllers, particularly those using optogenetics, face scalability challenges in industrial settings. In contrast, embedded and multicellular biomolecular controllers integrate the feedback control loop directly within the cellular machinery, using biological components. This design eliminates the need for external intervention and its associated limitations, making it a promising solution for scalable and adaptable control in various applications.

Different designs have been proposed to implement biomolecular feedback control strategies both *in silico* and *in vivo*, ranging from simple feedback and feedforward loops to PID (Proportional Integral Derivative) controllers (see ref 3 and references therein). In particular, the introduction of an integral control action *via* an antithetic motif has been highlighted as a fundamental ingredient for a synthetic circuit able to achieve and maintain a desired output even in the presence of disturbances, a property that has been termed *robust perfect adaptation*.^7^ Integral biomolecular controllers have been thoroughly studied both theoretically^8–10^ and experimentally, and different implementations have been proposed in bacteria^11^ and mammalian cells.^12^ For a review of the recent advancements in the implementations of biomolecular controllers guaranteeing robust perfect adaptation see ref 4.

However, existing robust controllers predominantly function at the single-cell level, posing inherent limitations on circuit complexity due to factors such as competition for limited resources^13^ or incompatible chemical reactions, and hindering their application as flexible plug-and-play modules across diverse synthetic and endogenous systems.^5^ To overcome these constraints, following ref 14 we propose to distribute the required functionalities across distinct cell populations within a microbial consortium. Each cell strain embeds specific engineered gene networks, forming the foundation for a distributed feedback control loop^15,16^ involving two populations: a controller population and a target cell population. The former senses the targets’ output, compares it with a reference signal encoding the desired expression level, and provides the latter with stimuli based on antithetic control logic so as to steer the sensed output toward the desired goal.^14,17,18^

Our work builds upon the foundation of synthetically engineered microbial consortia, where microbial cooperation, segregation, and division of labor have been leveraged for applications ranging from complex compound production^19^ to advanced computational processes^20^ and population density control^21^ (refer to refs 22–24 for a comprehensive review). Despite these advancements, as highlighted in ref 4, the *in vivo* implementation of synthetic communities implementing a distributed feedback control loop for the robust regulation of a desired phenotype to a specific reference value remains unexplored. Prior studies, such as Shou’s,^25^ have established mutualistic interactions between two yeast populations for coexistence, and research documented in ref 26 achieved synchronized oscillations *via* an engineered feedback loop in two populations derived from the same parent strain. Furthermore, a majority sensing mechanism in a microbial consortium composed by two populations derived from the same parent strain was realized through negative feedback in ref 27. While these studies successfully created synthetic microbial consortia with targeted phenotypes, they lack the capability for precise and robust tuning of expression, a critical aspect for advanced synthetic biology applications.

In this paper, we address this gap in the literature by developing and validating *in vivo* a feedback control loop distributed across a bacterial consortium, in which a controller population is tasked to regulate the expression of a gene hosted within a second population called targets. This is achieved by designing two populations embedding all the modules required to implement the three fundamental functions needed for control, namely computation, sensing and actuation; a strategy first proposed in silico in ref 14. We show that the designed cellular populations respond to their respective inputs and that the two populations can exchange information through two rationally designed communication channels, implemented using orthogonal quorum sensing signaling molecules. To demonstrate the validity of the proposed strategy, we carry out *in vivo* experiments comparing the closed loop configuration, where the controller cells can sense the signaling molecules from the targets, with an open loop implementation, where they cannot. Our results demonstrate that the proposed multicellular control architecture successfully achieves reliable and robust gene expression regulation in the target population, maintaining robustness to composition imbalances in the consortium—an essential feature for practical applications.

## Results

### Multicellular Distributed Control Architecture

The implementation of a multicellular control architecture requires the rational design of two populations, namely the *controllers* and the *targets* (see Figure 1a). The controllers receive information about the targets’ output (*y*), which is the expression level of some gene of interest therein. This output is compared to a reference signal (*ref*) encoding the desired expression level, and a control input (*u*) is then generated *via* the designed control logic.

**Figure 1:**
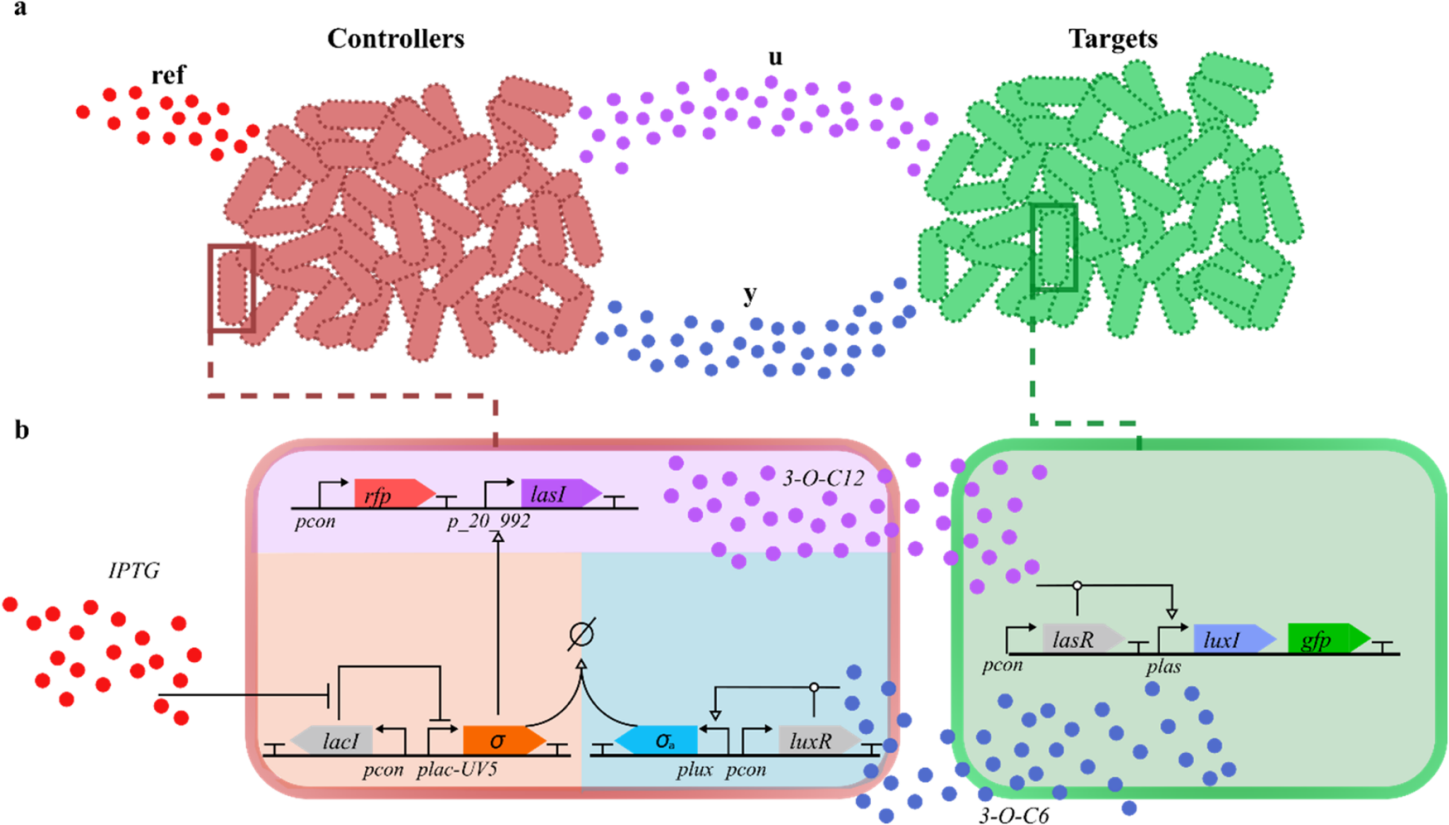
Multicellular control schematic. (a) Microbial consortium implementing a multicellular control architecture. The two populations, *i.e.*, controllers and targets, share information using orthogonal quorum sensing channels (**u** and **y**), playing the role of the control input and the output of the process, respectively. In addition, the controllers sense the external stimulus labeled as **ref**, used to set up the reference of the feedback loop. (b) Biological implementation of the multicellular control architecture. The Synthetic Biology Open Language (SBOL) notation is used to denote promoters, genes, promotion/inhibition relationships and quorum sensing molecules. The shaded areas identify different functional modules within each population.

The control input generated by the controllers is transmitted to the targets to regulate their output to the level encoded by the reference signal. The communication between controllers and targets is implemented *via* a pair of Quorum Sensing molecules (3–O–C6-HSL and 3–O–C12-HSL). The choice of these two molecules was guided by the compatibility of their operation ranges (*i.e.*, the ranges where the input-output response does not exhibit saturation). Additionally, as discussed in Section S4, they can be considered orthogonal in our design. Each cell population produces signaling molecules that can diffuse through the growth environment and into the other population, enabling effective cross-population communication.

The control logic, embedded in the controllers, is an antithetic feedback control strategy,^3^ based on the *in silico* analysis presented in ref 14 and derived from the error computation module (comparator) implemented *in vivo* using molecular titration as shown in ref 28 and characterized dynamically in ref 29. The controller network functions as a multi-input, single-output device, using two independent signals to regulate the expression level of its output protein (see Figure 1b).

The controller is built around a σ factor,^30^ a protein that recruits RNA polymerase (RNAP) to target promoters by binding to DNA sequences in the −10 and −35 regions of those promoters. Its corresponding anti-σ factor blocks this interaction by sequestering the σ factor, preventing RNAP from binding. The production of the σ factor is controlled by IPTG (the reference signal), which induces expression from the promoter *plac-UV5*, while the production of the anti-σ factor is regulated by the *plux* promoter, activated by the LuxR/3–O–C6-HSL complex, where LuxR is produced constitutively, and 3–O–C6-HSL is produced by the target cells.

The output signal from the controller cells (3–O–C12-HSL) is produced by the product of the *lasI* gene, regulated by the *p20_992* promoter. This promoter, placed upstream of *lasI*, is induced by the orthogonal σ factor. The σ and anti-σ factors interact by forming a complex that cannot recruit RNAP to the *p20_992* promoter. To prevent excessive expression levels of σ and anti-σ factors, which persisted even without external stimuli, we engineered both proteins with a degradation tag (ssrA tag).^31^ This modification, already discussed in refs 28,29 as a tool to improve the response time of the circuit, was essential for achieving an output from the controllers that was within the compatible input range of the targets, as detailed in the Gene Expression Can be Regulated *via* a Multicellular Architecture section.

Although the degradation tags accelerate protein degradation, potentially complicating perfect integration, previous studies have shown that antithetic motifs with “leaky integration” still provide satisfactory static and dynamic performance in closed loop configurations.^32^ This approach also prevents the accumulation of controller molecules which could interfere with the cells’ physiological functions.

Our numerical simulations, detailed in Section S3, demonstrate that the inclusion of degradation tags enhances the overall robustness of the system. Specifically, the system is able to maintain its desired performance across a broader range of conditions, ensuring stable operation even in the face of environmental fluctuations. This added robustness is crucial for practical applications where stability is a priority. In summary, the incorporation of degradation tags strikes a balance between maintaining control performance and safeguarding cellular health, resulting in a more robust and stable system overall.

The target population is designed to express green fluorescent protein (GFP), whose expression level is the variable we aim to regulate. The *gfp* gene is cotranscribed with *luxI*, whose product catalyzes the production of the molecule 3–O–C-6-HSL. This small molecule serves as a proxy of the *gfp* expression levels by diffusing in the growth environment and activating the production of anti-σ in the controllers’ population. The expression of LuxI and GFP is regulated by the *plas* promoter, which is activated by the LasR/3–O–C12-HSL complex. LasR is constitutively expressed, while 3–O–C12-HSL is produced by the controller cells. To ensure rapid degradation, LuxI was fused to a degradation tag (ssrA tag). Further details regarding the plasmids and strains used in this study can be found in the “Strains and Constructs” section of the Methods.

The goal of the design is to enable the two engineered populations to cooperate in steering and maintaining *gfp* expression in the targets at a desired set point, which can be adjusted by modifying the concentration of IPTG. Specifically, when *gfp* is overexpressed, the excessive production of 3–O–C6-HSL leads to an upregulation of anti-σ production. This, in turn, reduces the availability of free σ, which downregulates the production of LasI and consequently the production of the signaling molecule 3–O–C12-HSL. As a result, the activity of the *plas* promoter in the target population decreases, lowering *gfp* expression levels. Conversely, when *gfp* expression falls below the desired level, similar mechanisms work to increase GFP production to restore the set point.

### Gene Expression Can Be Regulated *via* a Multicellular Control Architecture

Prior to mixing the two populations in the consortium, we tested the functional modules embedded in each population using flow cytometry.^33^ Details of the machines and the settings used can be found in the Preparation of Samples and Analysis Using Flow Cytometry section of the Methods.

We started by characterizing the response of the controller population to IPTG and 3–O–C6-HSL, which serve as the reference signal and proxy for the target’s state, respectively. For this characterization we replaced *lasI* with *gfp* fused with an ssrA tag (see Figure S1) and removed the red fluorescent tag from the controllers. This characterization strain was intentionally designed as a preliminary, simplified circuit aimed at verifying the input-response of the controller population. The controllers were grown at 37 °C for 6 h in media supplemented with different concentrations of IPTG and 3–O–C6-HSL to test their response. Fluorescence levels were analyzed by flow cytometry (for details, see the “Induction Protocol” section of the Methods).

The addition of IPTG induced *plac-UV5* activity by inhibiting the repression from LacI, resulting in up to a 50-fold increase in steady-state fluorescence when 100 μM IPTG was added (Figure 2a). In contrast, adding 3–O–C6-HSL increased the anti-σ levels by activating *plux*, leading to a consistent decrease in fluorescence levels. This resulted in the inhibition of GFP levels by up to 50-fold when 100 nM 3–O–C6-HSL was introduced (Figure 2a).

**Figure 2:**
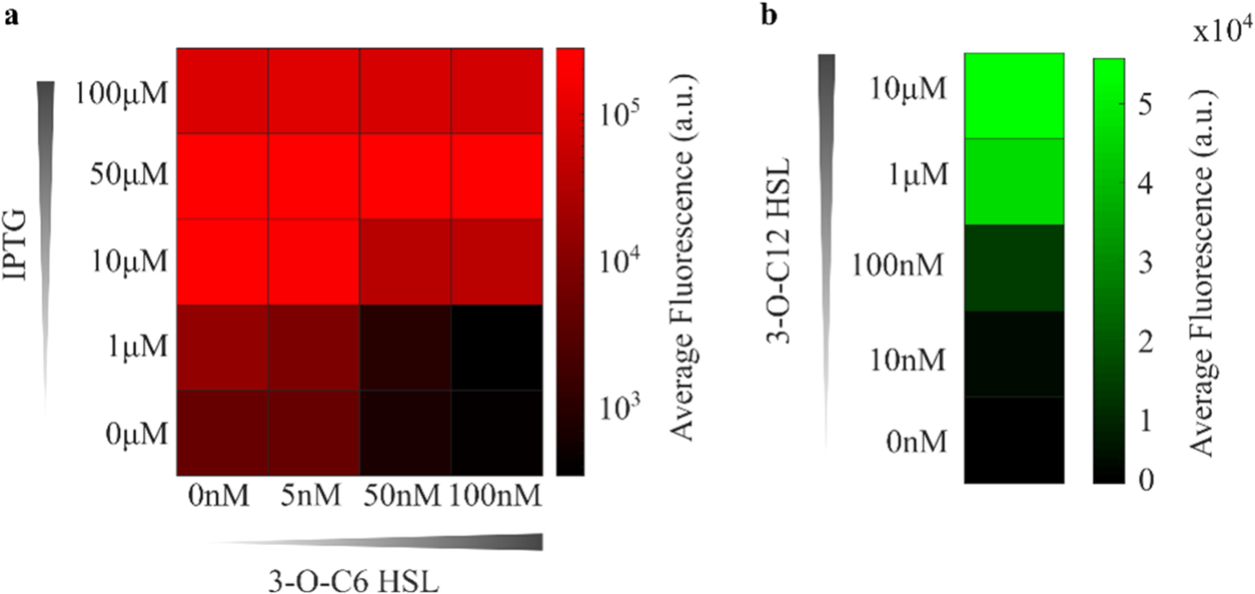
Characterization of the controllers and targets cell populations. To measure the output level of the controllers, we substituted *lasI* with *gfp* fused to an ssrA tag (Figure S1). (a) Average fluorescence of the controller population where the *lasI* gene was substituted with a *gfp*, as explained in the Gene Expression Can Be Regulated *via* a Multicellular Architecture section. This population was induced using different concentrations of 3–O–C6-HSL and IPTG. The data were collected after 6 h from the initial inoculation. (b) Average fluorescence levels of the target population induced using 3–O–C12-HSL. The data were collected after 6 h from the initial inoculation.

We followed a similar procedure to assess the response of the targets to 3–O–C12-HSL. As expected the addition of 10 μM 3–O-C12 to the growth media activated *plas*, leading to a 30 fold increase in average GFP fluorescence (Figure 2b). These assays confirmed that the controllers appropriately responded to changes in the reference signal or in the output of the targets, and that the targets could adjust GFP production according to the concentration of 3–O–C12-HSL. For further details on the standard deviation of the response of targets and controllers see Figure S2.

Next, we tested the ability of the controllers to regulate the expression of GFP in the target population. Specifically, we grew controllers and targets in coculture at 37 °C for 6 h (growth curves reported in Figure S3), both with and without IPTG in the growth medium and then analyzed the fluorescence level of the targets using flow cytometry. When the consortium was induced with 50 μM IPTG, the relative increase in fluorescence was approximately 50% with respect to the condition with no IPTG added to the growth medium (Figure S4c). Our results showed that the controllers could regulate the expression of GFP in the targets even in the absence of IPTG (for more details see Section S1).

However, the dynamic range of regulation was notably smaller than expected, based on the preliminary characterization of the populations. At the 6-h time point of the closed-loop experiment without IPTG (Figure S4), a comparison with the uninduced target response from Figure 2b revealed that the targets exhibited high fluorescence levels when mixed with the controllers, even when no IPTG was added to the culture media. Figure S5 further illustrates a comparison of the fluorescence distribution in the two scenarios. This observation suggests an unexpectedly high basal production of 3–O–C12-HSL, which was not apparent during the initial characterization of the populations.

To address the issue of high basal production, we reduced the synthesis rate of σ in the absence of IPTG to broaden the range of GFP fluorescence levels that could be regulated in the targets. Specifically, we increased the repression efficiency of *plac-UV5* by LacI by adding an extra *lac* operator upstream of the promoter. As shown in ref 34, this modification reduces basal gene expression by enabling the LacI tetramer to bind two operators simultaneously, thereby increasing the affinity of the interaction (see the “Strains and Constructs” section of the Methods for more details on plasmid construction). From this point onward, unless otherwise noted, the term “controllers” or “open-loop controllers” refers to this modified version with the extra lac operator on the σ plasmid.

After adding the extra operator, the steady-state fluorescence of the targets in the closed loop was reduced to levels lower than those reached by uninduced target monocultures. This can be observed by comparing the 0 h and 6 h time points in Figure 3c, where no IPTG was added to the growth media. The 0h time point corresponds to targets freshly mixed with the controllers, resembling a target monoculture, while the 6 h time point represents the steady-state fluorescence of the targets in the closed loop. This comparison indicates that the extra operator significantly reduced the leakiness of the *plac-UV5*promoter.

**Figure 3:**
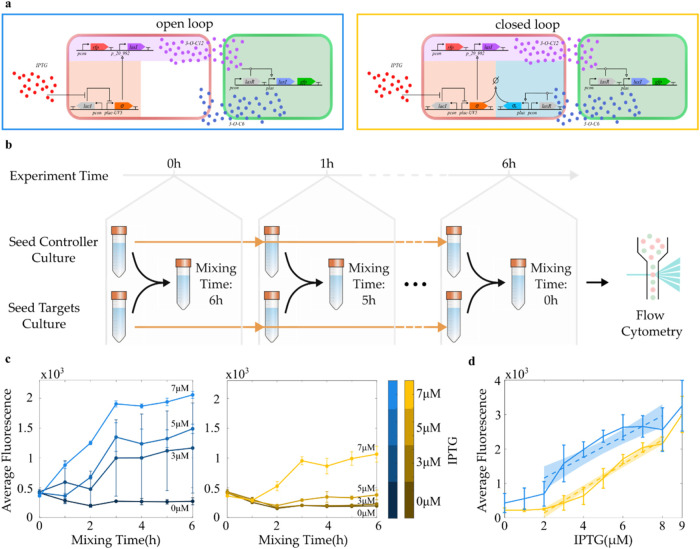
Regulation of the targets fluorescence using open and closed loop control using controllers/open loop controllers. (a) Schematic representation of the open loop (left) and the closed loop (right) configurations. (b) Schematic representation of the experimental protocol described in the Time-Course Assays section of the Methods. (c) Average fluorescence of the targets over a 6 h time course, using a closed (right panel) and open (left panel) loop control architecture with 0, 3, 5 and 7 μM *IPTG*, respectively. The solid dots represent the mean and the vertical bars the standard deviation of the data over *n* = 3 biological replicates. The lines are color coded with respect to the concentration of IPTG used. (d) Average steady state fluorescence of the targets over different IPTG concentrations. The blue (open loop) and yellow (closed loop) vertical bars are centered on the average values and their amplitude represents the standard deviation of the data over *n* = 3 biological replicates. The dashed lines represent the linear interpolation of the data obtained with IPTG concentrations in the range [2, 9 μM]. The shaded areas represent the confidence intervals of the linear model prediction. All steady states are collected after 6 h from the initial inoculation.

We then performed a dynamic characterization of the architecture using the protocol depicted in Figure 3b (more details are available in the Time Course Assays section of the methods). In these experiments all cultures were sampled after a total incubation time of 6 h. The time points shown in Figure 3c,3d represent the period during which controllers and targets were mixed (Mixing time). This method is similar to taking hourly samples from a coculture, with the advantage that every sample reaches a similar final density. To test the ability of the controllers to stimulate the targets without feedback from the latter, we implemented an open loop configuration of the consortium by removing the output sensing module from the controller cells (see Figure 3a). These *open-loop controllers* were unable to respond to feedback sensing molecules from the targets. This characterization showed that the open-loop controllers could stabilize *gfp* expression levels in the targets within 3 h. In addition, by increasing the IPTG concentration in the culture media, we could achieve up to a 10-fold increase in *gfp* expression levels. However, the regulation was highly variable across different biological replicates, especially at 3 μM IPTG (see Figure 3c).

When we closed the feedback loop by reintroducing the output-sensing module in the controllers, we observed improved regulation reliability. To quantify this improvement, we compared the Coefficient of Variation (CV) at each time point collected. At the 6 h time point, the CV decreased 5-fold at 0 μM IPTG and 6-fold at 3 μM IPTG compared to the open-loop configuration (see Figure S6). Conversely, the variability of the open loop architecture was lower with respect to the closed loop configuration at higher IPTG concentrations (5 and 7 μM). This effect is due to the insurgence of saturation in the open loop configuration, which constrains the range of possible responses and artificially reduces variability measures (see Supporting Figure S7 for further details and Section S2 of the Supporting Information for protocol details). The settling time remained comparable to the open-loop configuration (around 3 h), and we achieved up to a 5-fold increase in *gfp* expression at 7 μM IPTG (see Figure 3c). Although the closed-loop configuration resulted in a lower maximal induction compared to the open-loop system, the enhanced precision of regulation is a significant advantage. The feedback allows for more precise control over gene expression, which is critical in applications requiring consistent expression levels for optimal system performance or in environments with fluctuating conditions. This precision ensures that the desired gene is expressed at more consistent and reliable levels, reducing variability that could otherwise negatively affect downstream processes.

We further investigated the tunability of the regulation in both configurations by analyzing the steady state response with increasing levels of IPTG (details of the protocol used can be found in the Titration Assays section of the Methods). Specifically, we mixed the controllers or open-loop controllers with the targets and grew the consortium for 6 h at 37 °C. As shown in Figure 3d, both consortia were able to regulate the *gfp* gene to different expression levels, with both architectures showing saturation at IPTG concentrations within the range [0, 2 μM]. Beyond this range, higher IPTG concentrations led to increased fluorescence levels in the targets. Additionally, both configurations experienced an abrupt rise in average fluorescence and its variance in the targets at IPTG levels above 8 μM. Note that saturation effects, caused by the strongly nonlinear nature of the interaction between transcription factors, quorum sensing molecules and promoters, are well-known in the literature.^28,35^ However, in the in the context of gene circuits, these phenomena are undesirable. As such, characterizing the range of input signals capable of inducing changes in the output is crucial for defining the working conditions of the architecture.

Excluding saturation effects below 2 μM, we observed some distinct regulation patterns between the two configurations. The closed loop system displayed an almost linear increase in GFP fluorescence, whereas the open-loop system showed a sharp increase between 2 μM and 3 μM IPTG, followed by much flatter rise when IPTG concentrations exceeded 5 μM. To quantify the linearity of each configuration’s input-output response to IPTG, we fit the data using linear least-squares estimators and compared the *R* ^2^ values. In the closed-loop data (yellow dashed line in Figure 3d), the estimator nearly perfectly captured the variance of the data (*R* ^2^ = 0.91). Conversely, in the open-loop configuration, the fit accounted for just over half of the variance (*R* ^2^ = 0.67).

We also calculated the normalized mean squared error (NMSE) for both open- and closed-loop systems. The closed-loop system showed a 2-fold improvement, with an NMSE of 50, compared to the open-loop system’s NMSE of 106, confirming a significant improvement in the quality of the linear fit. This increased linearity in the closed-loop response results in a more predictable and stable gene expression profile, which is advantageous in industrial bioproduction settings where consistency is essential. By reducing deviations from expected expression levels, the closed-loop system can optimize yields and minimize waste, further demonstrating its superiority over the open-loop approach.

For the sake of completeness, we also characterized the input output response of this version of the controllers to IPTG by substituting *lasI* with *gfp*, removing the red fluorescent tag, and culturing controllers in LB monocultures supplemented with different IPTG concentrations. As shown in Figure S8, this characterization did not show any significant *gfp* expression at the levels of IPTG used in the control experiments (*i.e.*, IPTG ∈ [0 μM, 9 μM]). This experimental evidence highlights the difference in the levels of inductions needed for *lasI* to effectively regulate GFP fluorescence in the targets, and the *gfp* expression level within the controllers necessary for any detection using flow cytometry. More precisely, it suggests that the *lasI* expression level required to control GFP fluorescence in the targets is significantly lower than the GFP concentration needed for fluorescence detection in controllers where *lasI* was replaced with *gfp*.

Our experiments demonstrated the effectiveness of a multicellular control architecture in regulating the expression of a target gene within the population. Additionally, our experimental data show that incorporating feedback offers significant advantages over an open-loop configuration. Specifically, although feedback reduces the maximum induction possible for the target population, it improves regulation precision and enhances the linearity of the system’s response to different reference points.

### Closed Loop Control Enhances Robustness to Changes in the Consortium Composition

A key challenge in deploying multicellular strategies across a consortium of multiple cellular populations^36^ is that different populations may grow at different rates because of differences in their metabolic loads. This can lead to imbalances in the relative numbers of controllers and targets, impacting the controllers’ ability to regulate the targets’ dynamics by causing fluctuations in the production of sensing (3–O–C6-HSL) and actuation (3–O–C12-HSL) molecules. In our system, the total amount of the signaling molecules produced is directly proportional to the cell count within the consortium, assuming a constant IPTG concentration. This relationship is supported by the mathematical models detailed in refs 27,36,37 , where, supported by experimental observations, the total amount of quorum sensing molecules produced is modeled as being proportional to the number of cells in the population. Consequently, when the total cell count is held constant, an increase in the proportion of controller cells results in a higher total concentration of 3–O-C12 detected by the target population. Without feedback mechanisms, this would lead to increased fluorescence expression in the targets.

Conversely, when feedback is in place, a rise in the production of 3–O-C12 due to a higher number of controllers triggers an elevated expression of 3–O-C6. This, in turn, reduces 3–O-C12 production within the controllers. Hence, this feedback loop stabilizes *gfp* expression levels, demonstrating the system’s capacity to regulate target gene expression despite fluctuations in the proportion of controller cells within the consortium.

We analyzed both the closed loop and the open loop in consortia with different compositions to test the robustness of our multicellular feedback regulation against changes in the consortium composition­(see Figure 4). Specifically, we created consortia with different ratios of targets and controllers/open loop controllers in different ratios, then measured the average fluorescence of the targets after 6 h (for more detail see Consortium Composition Assay section in the Methods). For each replicate the data were normalized to the average expression of *gfp* reached in closed loop in the replicate. The normalization allowed us to investigate the robustness to population imbalances independently of the average steady-state level reached in each experimental replicate. This aspect is especially important in the context of industrial processes, as they typically operate based on relative stability rather than absolute output levels.

**Figure 4:**
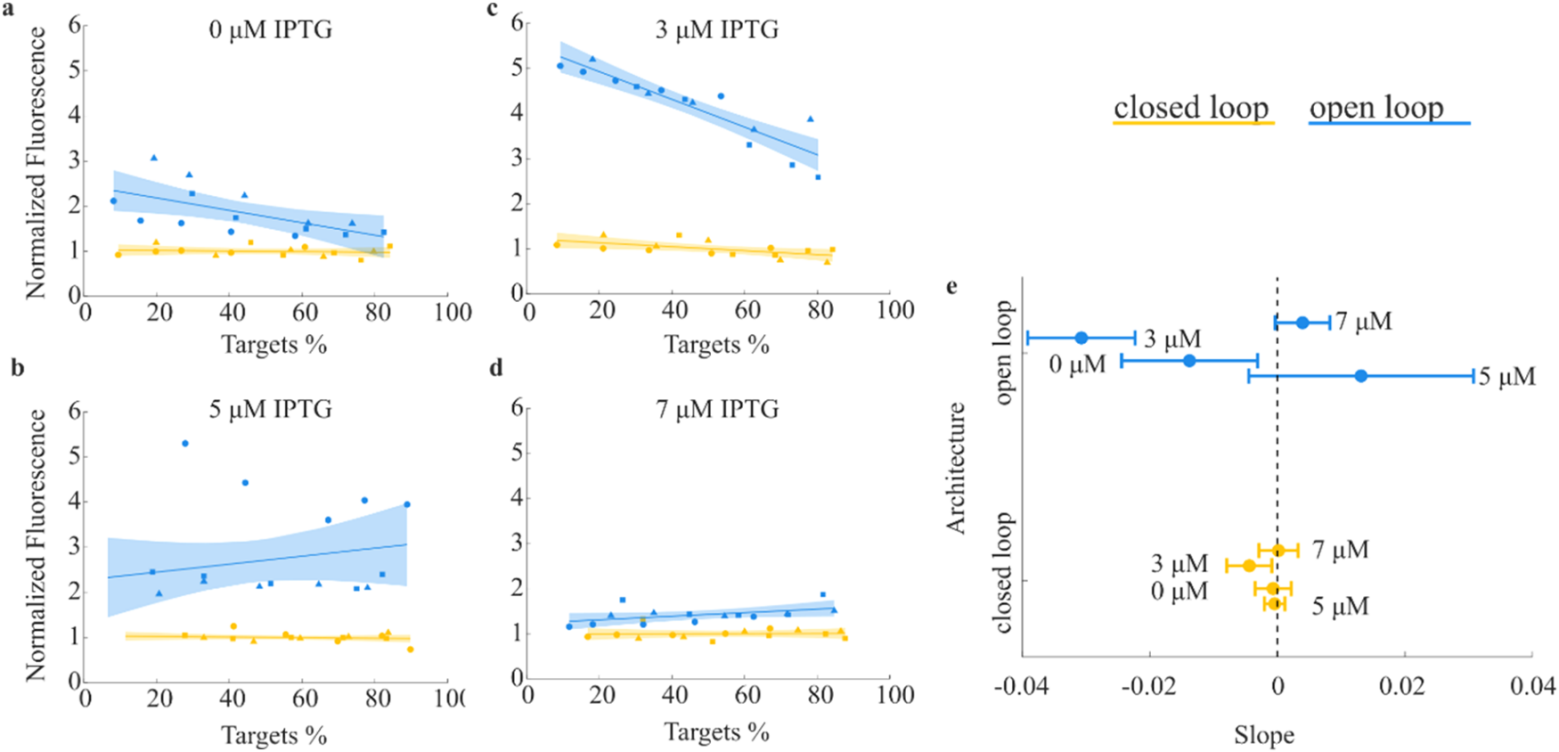
Closed loop control enhances robustness to changes in consortium composition. (a–d) Normalized average fluorescence in open (blue) and closed (yellow) loop across different consortium compositions using controllers/open loop controllers. The percentage of targets and the average fluorescence shown are measured after 6 h from the initial inoculation. The solid dots are the data collected over *n* = 3 biological replicates. Data points with the same shape belong to the same replicate. The data were fitted with a first order polynomial. The fitting and its confidence intervals are represented with the solid lines and the shaded areas, respectively. For each replicate, the data are normalized to the average fluorescence reached in closed loop across all compositions. (e) Comparison of the estimates of the slopes of the linear fittings of data in panels (a–d) when a closed (yellow) or open (blue) loop architecture was used. The solid dots represent the slope estimates and the horizontal bars the 90% confidence interval on the estimates. The non-normalized data are shown in Figure S9.

As shown in Figure 4a–d, varying the proportion of controllers and targets did not affect the reliability of regulation in the closed-loop configuration, where feedback between the two populations is present. In contrast, in the open-loop configuration (without feedback), we observed a decreasing trend in the fluorescence levels of the targets as the proportion of targets increased, particularly at IPTG concentrations of 0 and 3 μM. This trend was not significant when the IPTG concentration was 5 or 7 μM.

To quantify the effect of consortium composition on target fluorescence, we used linear least-squares estimators to fit the data (lines in Figure 4a–d) and compared the slopes of the lines for open- and closed-loop configurations at each IPTG concentration (see Figure 4e). Using analysis of covariance and a multiple comparison test, we found statistically significant differences in the slopes of linear models for closed and open-loop data points at 0 and 3 μM IPTG with *p*-values equal to 0.01527 and 1.13 × 10^–6^, respectively. However, at 5 and 7 μM IPTG, the slope differences were not significant. This outcome may be due to saturation of *plas* promoter activity at higher IPTG levels, reducing the targets’ sensitivity to changes in 3–O–C12-HSL concentration (see Supporting Figure S7 for further details and Section S2 of the Supporting Information for protocol details).

To complete our analysis, we conducted a statistical comparison of the slopes of the curves fitted to the closed-loop data points across all IPTG concentrations. Using the same methodology, we found no significant differences in slopes within the closed-loop system (detailed p-values provided in Table S3), confirming the robustness of the multicellular feedback control strategy in maintaining stability, even with imbalances between the controller and target populations.

To further support these findings, we derived a mathematical model to analyze steady-state behavior, examining the relationship between the steady-state fluorescence expressed by the target population and its proportion within the consortium. Our analysis revealed a linear dependence of fluorescence on the target population’s percentage in the open loop configuration. In contrast, the closed-loop model predicted a hyperbolic dependence, suggesting that, with suitable parameter selection, the influence of target cell proportion is considerably reduced in the closed-loop setup compared to the open-loop architecture (for further details see Section S3 and Figure S10). However, as this model does not account for saturation effects, it could not fully capture the qualitative shift in the open-loop response when IPTG ∈ [5, 7 μM].

Additionally, we numerically investigated the effects of enzymatic degradation by relaxing the assumption that dilution alone accounts for degradation of σ, anti-σ and 3–O–C12 HSL. In this scenario (Figure S11 and Section S3.3), we observed a reduced influence of the percentage of targets on the steady state expression level of *gfp* as the strength of enzymatic degradation increased. This finding underscores the role of degradation tags in enhancing the robustness of the architecture in both open- and closed-loop configurations.

## Discussion

Our study provides the first *in vivo* implementation of a biomolecular feedback controller for the regulation of gene expression to precise and tunable levels. This was achieved utilizing a multicellular control architecture in which a designated population of controller cells senses and regulates targeted phenotypes within another cell population. This strategic distribution of control functions across a microbial consortium alleviates the metabolic burden that would arise if all functions were confined to a single cell, as previously noted in the literature.^38^ Additionally, this architecture enhances the consortium’s robustness, allowing it to adapt more effectively to shifts in its composition.

After validating each component of our microbial community, we rigorously evaluated the overall performance and robustness of the architecture using flow cytometry. Our findings demonstrate that the distributed feedback controller proposed can reliably regulate gene expression in target cells, maintaining desired levels even when the composition of the consortium changes. To further substantiate the effectiveness of our control system, we conducted a comparative analysis between open- and closed-loop implementations. This comparison highlights the critical role of feedback mechanisms, showing that their inclusion substantially enhances both the reliability and robustness of gene regulation within the consortium.

The development of distributed feedback controllers across microbial communities paves the way for the construction of complex interacting communities, where each population carries out a specific function.^24^ Implementation of our distributed consortia within microfluidics/microscopy platforms would allow the realization of more complex feedback control tasks in space and time.^29,39,40^ This advancement could support applications ranging from engineering bacteria in the human gut microbiome to treat specific diseases,^41^ enhancing soil microbiomes to improve plant growth and health,^42^ to developing bacteria-based systems for biofuel production, which offers promising pathways for sustainable energy.^43^

Future work will focus on expanding the consortium by introducing additional controller populations to improve performance and robustness. For instance, incorporating populations capable of implementing proportional or derivative control actions could achieve a multicellular implementation of biomolecular PID controllers, as recently proposed in refs 8,17,44 .

## Methods

### Strains and Constructs

 TOP10 was used for all the cloning manipulations of this work. MG1655 λ-, rph-1 ^45^ was used for all the assays in this work and transformed with the plasmids constituting either the controllers or the targets. For all the sequences of the primers and the synthesized genes used in this study see Tables S1 and S2. In addition, all the maps of the plasmids are reported in Figure S12.

The controllers contain three plasmids: pVRa20_992_DS (medium copy number), pVRc20_992_BS1 (medium copy number) and pVRb_LasI (low copy number). The genes constituting the essential core of the computation module (σ, anti-σ and *lasI*) are ssrA tagged (AANDENYALAA) to ensure fast dynamics of expression and degradation.^31^

pVRb_lasI encodes the *lasI* gene, under the control of the *p_20992* promoter, and was built in ref 15, together with pVRb_ssrA, where the *lasI* gene was swapped with a superfolder *gfp*. pVRb_lasI was present in the controllers in each of the assays where they were mixed with the targets, whereas pVRb_ssrA was present in the controllers for their input output characterization.

Vector backbones for pVRa20_992_BS2 and pVRc20_992_BS1 were derived from plasmids pVRa20_992 and pVRc20_992.^30^ pVRa20_992_BS2 produces a σ factor under the control of a promoter that responds to IPTG. To construct this plasmid, first the pVRa20_992_BS1 plasmid was obtained by standard restriction digestion and ligation procedure, by amplifying the σ gene from the pLusB plasmid^28^ with primers Sigma_plus_tag_F and Sigma_plus_tag_R and cloning the PCR product in the pVRa_20_992 plasmid after NcoI/*Bam*HI digestion. Then, the *plac* promoter was substituted with *plac-UV5* to obtain the pVRa20_992_BS2 plasmid. The construct was built by standard restriction digestion and ligation procedure, by amplifying *plac-UV5* from the pVRa_placASb_Flag plasmid^28^ with primers PlacUV5_F and PlacUV5_R and cloning the PCR product in pVRa_20_992_BS1 plasmid after *XbaI* digestion. Finally, we modified pVRa_20_992_BS2 by introducing a second lac operator upstream of *plac_UV5*. This construct, denoted as PVRa_20_992_DS, was constructed by Gibson assembly after PCR amplification of pVRa20_992_BS2 using primers Back_extralac_for and Back_extralac_rev, and of the pVRbLacO1O1Del from^46^ using the Ins_extralac_for and Ins_extralac_rev primers. pVRc20_992_BS1 produces the anti-σ factor under the induction of 3–O–C6-HSL. The plasmid was constructed by standard restriction digestion and ligation procedure, by amplifying anti-σ from pVRa_placASb_Flag^28^ with primers Anti_Sigma_plus_tag_F and Anti_Sigma_plus_tag_R and cloning the PCR product in pVRc_20_992 plasmid after *Bam*HI/*Pst*I digestion. Finally, the controllers expressing a Red Fluorescent Protein were implemented by substituting the pVRb_lasI plasmid with the pVRb_lasI_RFP plasmid. pVRb_lasI_RFP was assembled using standard restriction digestion and ligation procedure, by amplifying a synthetically generated RFP with primers RFP_Afe_for and RFP_Nde_rev and cloning the PCR product in the pVRb_lasI plasmid after *AfeI/NdeI* digestion.

The targets hosted p_Las_Lux_GFP_3.0, a plasmid that embeds the 3–O–C12-HSL inducible promoter *plas*, driving the expression of a mut3 green fluorescent protein and of the luxI gene (fused with and ssrA tag). This plasmid was assembled by Gibson assembly after PCR amplification of the las_composite_device plasmid from ref 35 with p_Las_Lux_GFP_3_0_fwd_20 and p_Las_Lux_GFP_3_0_rev_20 primers, and of the luxI from pTeLu_GFP constructed in ref 28 with the luxI_fwd and luxI_rev primers.

### Chemicals

For all experiments, kanamycin (50 μg/mL), ampicillin (100 μg/mL) and chloramphenicol (25 μg/mL) were added to the growth media, depending on the bacteria selective resistance. These antibiotics were supplied by Sigma-Aldrich. pVRa_20992_BS2 and pVRa_20_992_DS have an ampicillin resistant gene, pVRb_lasI, pVRB_lasI_RFP and pVRb_ssrA have a gene conferring kanamycin resistance, and pVRc_20_992_BS1 and p_Las_Lux_GFP_3.0 have a gene guaranteeing chloramphenicol resistance. When multiple populations were mixed, the antibiotics to which both populations were resistant were used. If no resistance was in common, no antibiotic was used.

3–O–C6-HSL (N-(B-Ketocaproyl)-L-Homoserine Lactone from Sigma-Aldrich, cat # K3007) and 3–O–C12-HSL (N-(3-Oxododecanoyl)-L-homoserine lactone from Sigma-Aldrich, cat # O9139) were dissolved in DMSO, filter-sterilized and added to the LB growth medium at the indicated concentrations. IPTG (Isopropyl β-d-1-thiogalactopyranoside, supplied by Sigma-Aldrich, cat # I5502) was dissolved in water, filter-sterilized and added to LB medium at the indicated concentrations

### Preparation of Samples and Analysis Using Flow Cytometry

Each sample (0.5 mL volume) was resuspended in 1xPBS and diluted to achieve a final OD_600_ for the culture of 0.05. Data were acquired using either BD LSRFortessa X-20 or Acea NovoCyte flow cytometer. The results were analyzed using FlowJo.

The events recorded by a flow cytometer were first gated in the forward scatter (FSC-H, proxy of cellular size), side scatter (SSC-H, proxy of cellular complexity) plane. Here, healthy cells where size and complexity were in physiological ranges were selected^47^ (Figure S13a). Subsequently, healthy cells were gated in the SSC-H, SSC-A plane to select for single cells. Cells with comparable signals in the two channels were selected as single cells, whereas events with much larger SSC-A were classified as cellular conglomerates and excluded from the analysis (Figure S13b). Finally, single cells were gated in the PE-CF594 (560 nm excitation, 610/20 nm emission filter, Red) channel to discriminate targets and controllers based on their fluorescence levels. Specifically, cells with high PE-CF594 signal were classified as controllers and cells having low PE-CF594 fluorescence were selected to be targets (Figure S13c).

### Induction Protocol

Cells were cultured in 10 mL LB broth in 50 mL Falcon tubes. 20 μL of overnight cultures were added to the media, which was supplemented with the inducer(s) at the specified concentration. The culture was then incubated at 37 °C, shaking at 250 rpm for 6 h. Then, the samples were prepared and analyzed by flow cytometry. This protocol was used to obtain Figure 2a,b.

### Time-Course Assays

Controllers and targets populations were inoculated in 5 mL LB supplemented with the appropriate antibiotics at 37 °C, shaking at 250 rpm for approximately 13 h (overnight). Then, 35 μL of overnight cultures of controllers and 140 μL of overnight cultures of targets were separately diluted each in 35 mL LB added to a 250 mL sterile flask. Each culture was supplemented with the specified concentration of IPTG. This ratio between controllers and targets was empirically chosen such that the consortium at the end of the experiment comprised approximately half targets and half controllers (see Figure S3 for a representative example of the growth curves).

Both cultures were incubated at 37 °C, shaking at 250 rpm for 6 h. Each hour, 5 mL of both cultures were mixed in sterile 50 mL tubes, which were then incubated alongside the single strain cultures. After 6 h, 0.5 mL of all mixed cultures were sampled and analyzed *via* flow cytometry. Note that the hours in Figure 3c refer to the time targets and controllers have been mixed for, as shown in Figure 3b. Figure 3c was obtained using this protocol.

### Titration Assay

For each condition, 8 μL of overnight culture of targets and 2 μL of overnight culture of controllers were mixed in 10 mL LB broth in 50 mL Falcon tubes. Each culture was supplemented with IPTG at the specified concentration. The cultures were then incubated at 37 °C, shaking at 250 rpm for 6 h. Then, the samples were prepared and analyzed by flow cytometry. This protocol was used to obtain Figure 3d.

### Consortium Composition Assay

Overnight cultures of targets and controllers were diluted in 10 mL LB using different targets:controllers ratios. Specifically, 15:1, 8:1, 4:1, 2:1, 1:1 ratios were created by adding 10 μL of controllers and 150, 80, 40, 20, 10 μL of targets, respectively. All cultures were incubated at 37 °C shaking at 250 rpm for 6 h. Then, 0.5 mL of each culture was sampled and analyzed by flow cytometry. This protocol was used in Figure 4a–d. Note that all cultures reached stationary growth phase after 6 h, resulting in consortia with similar densities and different steady state percentages of Controllers and Targets.

## Supplementary Material



## Data Availability

The authors declare that the data supporting the findings of this study are available within the paper and its Supporting Information files or from the corresponding author on reasonable request. The source data underlying all figures in the main text and Supporting Information are provided as a Source Data file.

## Notes

Analysis of a preliminary version of the design (Sections S1); characterization of the sensitivity of the open loop architecture to exogenous addition 3–O–C12-HSL; (Section S2); mathematical modeling, formal analysis and numerical simulations of the robustness of the architecture to population variations; (Section S3); characterization of the orthogonality between the quorum sensing channels used (Section S4); tables reporting sequences of primers and synthetic genes used and statistical tests used for the robustness analysis; Figures representing the characterization strain, detailed characterization of the populations, performance of a preliminary multicellular architecture, characterization of the sensitivity of open loop to 3–O-C12, plasmid maps, flow cytometry gating strategy, unnormalized robustness data, numerical robustness analysis, growth curves, and characterization of orthogonality between quorum sensing molecules (PDF)
