# Supplementary material for: In Vivo Multicellular Feedback Control in Synthetic Microbial Consortia
Source: ACS Synth Biol. 2025 Jun 11;14(7):2537–47. doi: 10.1021/acssynbio.4c00862 (PMC12281617; doi:10.1021/acssynbio.4c00862)
Supplement: Supplementary file 1 [file sb4c00862_si_001.pdf]

SUPPLEMENTARY INFORMATION

In-vivo multicellular feedback control in synthetic microbial consortia

Davide Salzano[a,b], Barbara Shannon[b], Claire Grierson[c,d],

Lucia Marucci[d,e,f*], Nigel J Savery[b,d*], Mario di Bernardo [a,g,*]

[a]*Scuola Superiore Meridionale, 80134, Naples, Italy*

[b] *School of Biochemistry, University of Bristol, BS8 1TD, United Kingdom*

[c] *School of Biological Sciences, University of Bristol, BS8 1TQ, United Kingdom*

[d] *Bristol BioDesign Institute, University of Bristol, BS8 1QU, United Kingdom*

[e] *School of Engineering Mathematics and Technology, University of Bristol, BS8 1TW, United Kingdom*

[f] *School of Cellular and Molecular Medicine, University of Bristol, BS8 1TD, United Kingdom*

[g] *Department of Electrical Engineering and Information Technology, University of Naples Federico II, 80125, Italy*

* *These authors contributed equally*

[#] Correspondence should be addressed to: mario.di.bernardo@unina.it

Index

S1. Characterization of an earlier version of the architecture…………..…………………...….. 2

S2. Sensitivity analysis to 3-O-C12-HSL in open loop…………………………………...….. 3

S3. Mathematical modeling of the multicellular controller ..…………………………...….. 3

S4. Orthogonality between *las* and *lux* systems……………………………………………..12

SUPPLEMENTARY TABLES…………………………………………………………………… 14

SUPPLEMENTARY FIGURES……………………………..……………………………..... 17

S1. Characterization of an earlier version of the architecture.

After the preliminary characterization of the input-output response of controllers and targets to their respective inputs, we assayed the ability of the two populations to communicate with each other. To test the ability of the controllers to stimulate the targets in the absence of any feedback from the latter, we implemented an open loop configuration of the consortium where the output sensing module was removed from the controller cells (see Figure S4a, right panel). This made them unable to receive feedback sensing molecules from the targets.

We mixed the open loop controllers with the targets every hour, grew the consortium at 37 ^{o}C for 6h and analysed the targets fluorescence via flow cytometry (for further details on the experimental protocol see section *Time-course assays* of the methods). This time course, reported in Figure S4b (light blue trajectory), shows that the open loop controllers are able to communicate with the targets, i.e. that the 3-O-C12-HSL is successfully produced by the open loop controllers and sensed by the targets. Indeed, the controllers were able to induce a 15 fold increase in the average fluorescence of the targets after 6h. However, over this time period the open loop controllers were not able to stabilize the *gfp* expression levels in the targets confirming that, as expected, they were unable to receive any feedback signal sent from them. We performed similar experiments mixing the controllers with the targets. In closed loop, where controllers possess the module to sense the expression level of the controlled gene in the targets, stabilization of the targets fluorescence was observed after 3h (Figure S4b, yellow trajectory). This confirms that the targets were able to successfully produce 3-O-C6-HSL molecules which were correctly received by the controller population.

We then tested the ability of the controllers in the closed loop configuration to regulate *gfp* expression levels in the targets to different values by modulating the concentration of IPTG in the culture medium. Specifically, we grew controllers and targets in coculture at 37 ^{o}C for 6h, both with and without IPTG in the growth medium and then analysed the fluorescence level of the targets using flow cytometry. When the consortium was induced with 50 μM IPTG, the relative increase in fluorescence was approximately 50% (Figure S4c). This increase was surprisingly small compared to the expected increase in the output observed during the

preliminary characterisation of the populations. Comparing the 6h timepoint of the closed loop time course without IPTG shown in Figure S4b with the uninduced targets response shown in Figure 2b, we observed that the targets were expressing high levels of fluorescence when mixed with the controllers, even when no IPTG was present in the culture media (S4 shows a comparison of the distribution of fluorescence of the targets in the two scenarios). This suggests that there was an unexpected high basal production of 3-O-C12-HSL, which was not revealed during the initial characterisation of the populations shown in Figure 2a. The inability to reveal the base expression level in the initial characterisation highlights the difference between the expression level needed to detect any GFP signal and the levels of enzyme needed to produce enough signalling molecules to induce the targets.

Therefore, we reduced the synthesis rate of σ in the absence of IPTG, aiming to increase the range of GFP fluorescence levels we could regulate the targets to. Specifically, we increased the repression efficiency of plac-UV5 by LacI by adding an extra lac operator upstream of the promoter. This version of the controller will be denoted as *Controllers 2.0.* here.

S2. Sensitivity analysis to 3-O-C12-HSL in open loop

To assess the sensitivity of the *plas* promoter to 3-O-C12-HSL when the targets were mixed with the controllers in open loop, we added exogenous 3-O-C12-HSL to the consortium (see Figure S7). The results show no statistically significant change in the average fluorescence expressed by the targets when 5 μM and 7 μM IPTG were present. Instead, at lower IPTG concentrations, adding exogenously the actuation molecule caused a statistically significant increase in the fluorescence expressed by the targets. We also quantified the statistical relevance of the relative increase in target fluorescence levels when 1 nM 3-O-C12 was added to the media, confirming that at lower IPTG concentrations, i.e. 0 μM and 3 μM, the increase was statistically significant (p-values 0.0008, 0.01 for 0 μM, 3 μM IPTG using a t-test). Instead, at higher IPTG concentrations, the changes were found not to be statistically relevant (p-values 0.06, 0.12 for 5 μM, 7 μM IPTG using a t-test).

S3. Mathematical modeling of the multicellular controller

S3.1 Model derivation

Starting from the schematic design pictured in Figure 1, we derived an agent-based model obtained from the law of mass action kinetics and the polynomial model commonly used to

capture mathematically the antithetic sigma/antisigma interaction (see [1–3]). We denoted the concentration of a protein, say x, inside the i-th cell of the population as $\boldsymbol{x_i}$ with the vector $[\boldsymbol{x}]$ being the column vector whose components are the concentrations of protein x in each of the cells of the population. We defined $Z_1 = [\sigma]$, Z_2 =[anti-σ], Q_x =[3-O-C6-HSL], Q_u =[3-O-C12-HSL], X_T =[GFP], r = IPTG. Finally, we used the superscripts T, I, e to indicate the concentration of the quorum sensing molecules in the Target cells, Controller cells and in the external environment, respectively. Using this notation, we can write:

$$
\begin{aligned}
\dot{Z}_{1,i} &= \mu r - \gamma_z Z_{1,i} Z_{2,i} - \gamma Z_{1,i} \\
\dot{Z}_{2,i} &= \theta Q_{x,i}^{I} - \gamma_z Z_{1,i} Z_{2,i} - \gamma Z_{2,i} \\
\dot{Q}_{x,i}^{I} &= \eta\left(Q_x^e - Q_{x,i}^{I}\right) - \gamma Q_{x,i}^{I} \\
\dot{Q}_{u,i}^{I} &= \beta_I Z_{1,i} + \eta\left(Q_u^e - Q_{u,i}^{I}\right) - \gamma Q_{u,i}^{I} \\
\dot{X}_{T,i} &= \beta_T Q_{u,i}^{T} - \gamma X_{T,i} \\
\dot{Q}_{x,i}^{T} &= \beta_T Q_{u,i}^{T} + \eta\left(Q_x^e - Q_{x,i}^{T}\right) - \gamma Q_{x,i}^{T} \\
\dot{Q}_{u,i}^{T} &= \eta\left(Q_u^e - Q_{u,i}^{T}\right) - \gamma Q_{u,i}^{T} \\
\dot{Q}_x^e &= \eta \sum_{i=1}^{N_T}\left(Q_{x,i}^{T} - Q_x^e\right) + \eta \sum_{i=1}^{N_C}\left(Q_{x,i}^{I} - Q_x^e\right) \\
\dot{Q}_u^e &= \eta \sum_{i=1}^{N_T}\left(Q_{u,i}^{T} - Q_u^e\right) + \eta \sum_{i=1}^{N_C}\left(Q_{u,i}^{I} - Q_u^e\right).
\end{aligned}
\tag{3.1}
$$

Here μ and θ are production rates of $Z_{1,i}$ and $Z_{2,i}$, γ_z is the annihilation rate between $Z_{1,i}$ and $Z_{2,i}$, γ is the degradation rate due to cell growth and division, assumed to be the same for all chemical species. In addition, η is the diffusion rate of the quorum sensing molecules, β_I represents the gain of the controller and β_T is the activation rate of *gfp* and *luxI* induced by 3-O-C12-HSL. Note that we assumed that transcription happens much faster than translation (i.e. making a quasi-steady state assumption on the mRNA dynamics). For further details on the pseudo reactions used see [4,5].

To derive the aggregate model describing the evolution of the concentration of each chemical species at a population level, as done in [2,4,5], we assumed that the degradation due to growth and division of the species Z_1 and Z_2 (i.e. the terms $-\gamma Z_{1,i}$, $-\gamma Z_{2,i}$) is dominated by

the binding reaction and can therefore be neglected. Under this assumption, we can recast the model (3.1) as

$$
\begin{aligned}
\dot{Z}_{1,i} &= \mu r - \gamma_z Z_{1,i} Z_{2,i} \\
\dot{Z}_{2,i} &= \theta Q_{x,i}^{I} - \gamma_z Z_{1,i} Z_{2,i} \\
\dot{Q}_{x,i}^{I} &= \eta\left(Q_x^e - Q_{x,i}^{I}\right) - \gamma Q_{x,i}^{I} \\
\dot{Q}_{u,i}^{I} &= \beta_I Z_{1,i} + \eta\left(Q_u^e - Q_{u,i}^{I}\right) - \gamma Q_{u,i}^{I} \\
\dot{X}_{T,i} &= \beta_T Q_{u,i}^{T} - \gamma X_{T,i} \\
\dot{Q}_{x,i}^{T} &= \beta_T Q_{u,i}^{T} + \eta\left(Q_x^e - Q_{x,i}^{T}\right) - \gamma Q_{x,i}^{T} \\
\dot{Q}_{u,i}^{T} &= \eta\left(Q_u^e - Q_{u,i}^{T}\right) - \gamma Q_{u,i}^{T} \\
\dot{Q}_x^e &= \eta \sum_{i=1}^{N_T}\left(Q_{x,i}^{T} - Q_x^e\right) + \eta \sum_{i=1}^{N_C}\left(Q_{x,i}^{I} - Q_x^e\right) \\
\dot{Q}_u^e &= \eta \sum_{i=1}^{N_T}\left(Q_{u,i}^{T} - Q_u^e\right) + \eta \sum_{i=1}^{N_C}\left(Q_{u,i}^{I} - Q_u^e\right).
\end{aligned}
\tag{3.2}
$$

Additionally, defining $\zeta_i = Z_{1,i} - Z_{2,i}$ we can recast the dynamics of Z_1, Z_2 and Q_u^I in Equation (3.2) as

$$
\begin{aligned}
\dot{\zeta}_i &= \mu r - \theta Q_{x,i}, \\
\dot{Z}_{2,i} &= \theta Q_{x,i} - \gamma_z\left(\zeta_i + Z_{2,i}\right) Z_{2,i}, \\
\dot{Q}_{u,i}^{I} &= \beta_I Z_{1,i} + \eta\left(Q_u^e - Q_{u,i}^{I}\right) - \gamma Q_{u,i}^{I}
\end{aligned}
\tag{3.3}
$$

Defining $\epsilon = \frac{1}{\gamma_z}$, Equation (3.3) can be recast as

$$
\begin{aligned}
\dot{\zeta}_i &= \mu r - \theta Q_x, \\
\epsilon \dot{Z}_{2,i} &= \epsilon \theta Q_x - \left(\zeta_i + Z_{2,i}\right) Z_{2,i} \\
\dot{Q}_{u,i}^{I} &= \beta_I\left(\zeta_i + Z_{2,i}\right) + \eta\left(Q_u^e - Q_{u,i}^{I}\right) - \gamma Q_{u,i}^{I},
\end{aligned}
\tag{3.4}
$$

Using a singular perturbation theory argument and assuming $\epsilon \to 0$ (i.e. $\gamma_z \to \infty$) as done in [4], we can neglect the dynamics of $Z_{2,i}$. Computing its steady state we get

$$
\left(\zeta_i + Z_{2,i}\right) Z_{2,i} = 0 \tag{3.5}
$$

which admits as only admissible solution $Z_{2,i} = 0$. Substituting the solution of equation (3.5) into the equation (3.4) we can recast model (3.2) as

$$\begin{aligned}
\dot{\zeta_i} &= \mu r - \theta Q_{x,i}^I \\
\dot{Q}_{x,i}^I &= \eta\left(Q_x^e - Q_{x,i}^I\right) - \gamma Q_{x,i}^I \\
\dot{Q}_{u,i}^I &= \beta_I \zeta_i - \gamma Q_{u,i}^I + \eta\left(Q_u^e - Q_{u,i}^I\right) \\
\dot{X}_{T,i} &= \beta_T Q_{u,i}^T - \gamma X_{T,i} \\
\dot{Q}_{x,i}^T &= \beta_T Q_{u,i}^T + \eta\left(Q_x^e - Q_{x,i}^T\right) - \gamma Q_{x,i}^T \\
\dot{Q}_{u,i}^T &= \eta\left(Q_u^e - Q_{u,i}^T\right) - \gamma Q_{u,i}^T \\
\dot{Q}_x^e &= \eta \sum_{i=1}^{N_T}\left(Q_{x,i}^T - Q_x^e\right) + \eta \sum_{i=1}^{N_C}\left(Q_{x,i}^I - Q_x^e\right) \\
\dot{Q}_u^e &= \eta \sum_{i=1}^{N_T}\left(Q_{u,i}^T - Q_u^e\right) + \eta \sum_{i=1}^{N_C}\left(Q_{u,i}^I - Q_u^e\right).
\end{aligned} \tag{3.6}$$

Assuming that the number of cells in the consortium is fixed and that it is composed of N_T targets and N_C controllers, we can define the average concentration for each species as $X_T = \frac{1}{N_T}\sum_{i=1}^{N_T} X_{T,i}$, $Q_x^T = \frac{1}{N_T}\sum_{i=1}^{N_T} Q_{x,i}^T$, $Q_u^T = \frac{1}{N_T}\sum_{i=1}^{N_T} Q_{u,i}^T$, $\zeta = \frac{1}{N_C}\sum_{i=1}^{N_C} \zeta_i$, $Q_x^I = \frac{1}{N_C}\sum_{i=1}^{N_C} Q_{x,i}^I$, $Q_u^I = \frac{1}{N_C}\sum_{i=1}^{N_C} Q_{u,i}^I$. Note that the assumption of a constant number of cell is representative of a steady state condition of the consortium in batch cultures (for more details on growth modeling see section S3.4). We can then obtain the following model describing the evolution of such average concentrations as

$$\begin{aligned}
\dot{\zeta} &= \mu r - \theta Q_x^I \\
\dot{Q}_x^I &= \eta(Q_x^e - Q_x^I) - \gamma Q_x^I \\
\dot{Q}_u^I &= \beta_I \zeta - \gamma Q_u^I + \eta(Q_u^e - Q_u^I) \\
\dot{X}_T &= \beta_T Q_u^T - \gamma X_T \\
\dot{Q}_x^T &= \beta_T Q_u^T + \eta(Q_x^e - Q_x^T) - \gamma Q_x^T \\
\dot{Q}_u^T &= -\gamma Q_u^T + \eta(Q_u^e - Q_u^T) \\
\dot{Q}_x^e &= \eta N_T(Q_x^T - Q_x^e) + \eta N_C(Q_x^I - Q_x^e) \\
\dot{Q}_u^e &= \eta N_T(Q_u^T - Q_u^e) + \eta N_C(Q_u^I - Q_u^e).
\end{aligned} \tag{3.7}$$

Note that the time scale separation used to reduce the order of the model is fundamental to obtain the aggregate model without introducing other modeling assumptions (e.g. homogeneity of gene expression between cells of the same population) [4,6].

Following the same reasoning it is possible to derive the aggregate model for the open loop architecture, which can be formalized as

$$\begin{aligned}
\dot{Z}_1 &= \mu r - \gamma Z_1 \\
\dot{Q}_x^I &= \eta(Q_x^e - Q_x^I) - \gamma Q_x^I \\
\dot{Q}_u^I &= \beta_I Z_1 - \gamma Q_u^I + \eta(Q_u^e - Q_u^I) \\
\dot{X}_T &= \beta_T Q_u^T - \gamma X_T \\
\dot{Q}_x^T &= \beta_T Q_u^T + \eta(Q_x^e - Q_x^T) - \gamma Q_x^T \\
\dot{Q}_u^T &= -\gamma Q_u^T + \eta(Q_u^e - Q_u^T) \\
\dot{Q}_x^e &= \eta N_T(Q_x^T - Q_x^e) + \eta N_C(Q_x^I - Q_x^e) \\
\dot{Q}_u^e &= \eta N_T(Q_u^T - Q_u^e) + \eta N_C(Q_u^I - Q_u^e),
\end{aligned} \tag{3.8}$$

where $Z_1 = \frac{1}{N_C}\sum_{i=1}^{N_C} Z_{1,i}$. Note that here the assumption of neglecting the degradation of Z_1 cannot be made as the annihilation reaction is not present in the consortium.

S3.2 Steady state analysis

To gauge the regulation capabilities of the closed loop and open loop architectures, we computed the steady state expression of X_T. For the closed loop architecture, we started by imposing $\dot{Q}_X^e = 0$, obtaining:

$$Q_x^e = \frac{N_T}{N_C+N_T} Q_x^T + \frac{N_C}{N_C+N_T} Q_x^I. \tag{3.9}$$

If we define $\alpha = \frac{N_T}{N_C+N_T}$ and $1 - \alpha = \frac{N_C}{N_C+N_T}$, we can write:

$$Q_x^e = \alpha Q_x^T + (1-\alpha) Q_x^I. \tag{3.10}$$

Then, solving $\dot{Q}_X^I = 0$ by exploiting (3.10) we get

$$Q_x^I = \frac{\eta\alpha}{\gamma+\eta\alpha} Q_x^T\,. \tag{3.11}$$

Solving $\dot{Q}_X^t = 0$ and substituting its solution in (3.11) we obtain

$$Q_x^T = \frac{\beta_T}{\gamma}\frac{\gamma+\eta\alpha}{\gamma+\eta}Q_u^T,\ Q_x^I = \frac{\beta_T\eta\alpha}{\gamma(\gamma+\eta)}Q_u^T. \tag{3.12}$$

In addition, solving $\dot{\zeta} = 0$, we can write:

$$Q_x^I = \frac{\mu r}{\theta}. \tag{3.13}$$

Substituting equation (3.13) in equation (3.12) yields

$$Q_u^T = \frac{\mu r\gamma(\gamma+\eta)}{\theta\beta_T\eta\alpha}. \tag{3.14}$$

Finally, solving $\dot{X}_T = 0$ we get

$$X_T = \frac{\beta_T Q_u^T}{\gamma}. \tag{3.15}$$

which, substituting equation (3.14) can be recast as

$$X_T = \frac{\mu r(\gamma+\eta)}{\theta\eta\alpha}. \tag{3.16}$$

For the Open loop architecture we can start computing the steady state expression of Q_u^T and Q_u^I. Repeating the same process done for Q_x, we get

$$Q_u^I = \frac{\beta_I}{\gamma}\frac{\gamma+\eta(1-\alpha)}{\gamma+\eta}Z_1, Q_u^T = \frac{\beta_I\eta(1-\alpha)}{\gamma(\gamma+\eta)}Z_1. \tag{3.17}$$

Additionally, solving $\dot{Z}_1 = 0$ we obtain

$$Z_1 = \frac{\mu r}{\gamma}. \tag{3.18}$$

Finally, substituting equation (3.17) and (3.18) in equation (3.15) we can write

$$X_T = \frac{\mu r\beta_T\beta_I\eta(1-\alpha)}{\gamma^3(\gamma+\eta)}. \tag{3.19}$$

If we assume that the diffusion is much faster than the degradation (i.e. $\eta \gg \gamma$), we can simplify equations (3.16), (3.19) as

$$X_T^C = \frac{\mu r}{\theta\alpha},\quad X_T^O = \frac{\mu r\beta_T\beta_I(1-\alpha)}{\gamma^3}, \tag{3.20}$$

where the superscript C and O have been added to denote the expected closed loop and open loop concentrations of gfp at steady state, respectively. Denoting the desired steady state value for X_T to be $\frac{\mu r}{\theta}$, we can normalize equation (3.19), retrieving

$$\tilde{X}_T^C = \frac{X_T^C\theta}{\mu r} = \frac{1}{\alpha},\quad \tilde{X}_T^O = \frac{X_T^O\theta}{\mu r} = \frac{\theta\beta_T\beta_I(1-\alpha)}{\gamma^3}. \tag{3.21}$$

We quantified the influence of the consortium composition on the steady state *gfp* fluorescence by computing $\frac{d\tilde{X}_T^i}{d\alpha}, i \in \{C, O\}$. Comparing this derivative in open and closed loop, it is possible to show that for

$$\alpha > \sqrt{\frac{\gamma^3}{\theta\beta_T\beta_I}} \qquad (3.22)$$

the consortium composition on the steady state fluorescence is less influential in closed loop than in open loop.

This analysis highlights the difference between the nature of the relationship between the fluorescence expressed at steady state by the targets and their percentage within the consortium in open and closed loop. Specifically, in open loop we retrieve exactly the linear relationship observed in the experimental data. Instead, in the closed loop scenario the relation retrieved is not independent, but there is an hyperbolic scaling due to the imbalances in the population numbers between controllers and targets. To support this claim we simulated systems (3.1) in the open and closed loop configuration using parameters taken from [4] and compared the normalized steady state. From the results shown in Figure S10 it is possible to see qualitative differences in the regulation patterns in closed and open loop. For this choice of the parameters, the closed loop architecture shows an increased robustness for a wide range of consortium compositions ($\alpha \in [0.22,1]$).

Notice that this model does not include any saturation effects, therefore it is not able to capture differences between the behavior of the open loop architecture at low (0 µM, 3 µM) and high (5 µM, 7 µM) IPTG concentrations.

S3.3 Effects of enzymatic degradation on the robustness

The model presented in section S3 only partially captures the behavior observed experimentally. Although this simplified model is able to exactly capture the expected linear relation between the steady state fluorescence expressed in the Target population for low concentrations of IPTG, in the closed loop configuration the model predicts an hyperbolic relationship between steady state fluorescence and percentage of targets in the consortium, which was not observed experimentally.

To understand the factors influencing the robustness observed *in vivo* we expanded the model to include the effects of enzymatic degradation caused by the addition of ssrA tags. Specifically, we added an additional degradation term on the species involved in the molecular titration (i.e. Z_1 and Z_2), as well as the Quorum Sensing molecule Q_u^I. Also, we modeled the dynamics of the inert complex formed by Z_1 and Z_2, denoted as Z_{12}. With these additions, model (3.2) can be modified into

$$
\begin{aligned}
\dot{Z}_{1,i} &= \mu r - \gamma_z Z_{1,i} Z_{2,i} - \gamma Z_{1,i} - \frac{\gamma_d}{K_d + Z_{1,i} + Z_{2,i} + Z_{12,i} + Q_{u,i}^I} Z_{1,i} \\
\dot{Z}_{2,i} &= \theta Q_{x,i}^I - \gamma_z Z_{1,i} Z_{2,i} - \gamma Z_{2,i} - \frac{\gamma_d}{K_d + Z_{1,i} + Z_{2,i} + Z_{12,i} + Q_{u,i}^I} Z_{2,i} \\
\dot{Z}_{12,\mathrm{i}} &= \gamma_z Z_{1,i} Z_{2,i} - \gamma Z_{12,i} - \frac{\gamma_d}{K_d + Z_{1,i} + Z_{2,i} + Z_{12,i} + Q_{u,i}^I} Z_{12,i} \\
\dot{Q}_{x,i}^I &= \eta\left(Q_x^e - Q_{x,i}^I\right) - \gamma Q_{x,i}^I \\
\dot{Q}_{u,i}^I &= \beta_I Z_{1,i} + \eta\left(Q_u^e - Q_{u,i}^I\right) - \gamma Q_{u,i}^I - \frac{\gamma_d}{K_d + Z_{1,i} + Z_{2,i} + Z_{12,i} + Q_{u,i}^I} Q_{u,i}^I \\
\dot{X}_{T,i} &= \beta_T Q_{u,i}^T - \gamma X_{T,i} \\
\dot{Q}_{x,i}^T &= \beta_T Q_{u,i}^T + \eta\left(Q_x^e - Q_{x,i}^T\right) - \gamma Q_{x,i}^T \\
\dot{Q}_{u,i}^T &= \eta\left(Q_u^e - Q_{u,i}^T\right) - \gamma Q_{u,i}^T \\
\dot{Q_x^e} &= \eta \sum_{i=1}^{N_T} \left(Q_{x,i}^T - Q_x^e\right) + \eta \sum_{i=1}^{N_C} \left(Q_{x,i}^I - Q_x^e\right) \\
\dot{Q_u^e} &= \eta \sum_{i=1}^{N_T} \left(Q_{u,i}^T - Q_u^e\right) + \eta \sum_{i=1}^{N_C} \left(Q_{u,i}^I - Q_u^e\right).
\end{aligned}
\tag{3.23}
$$

Here, γ_d represents maximal rate of enzymatic degradation and K_d half-activation threshold of the degradation. Note that the enzymatic degradation was modeled as in [7].

We numerically investigated the effects of the degradation tag by simulating system (3.23). Specifically, we set all the parameters but γ_d to feasible values found in the literature and varied the value of γ_d, recording the steady state of X_T in dependence of the percentage of Targets in the consortium, both in open loop and in closed loop. In each numerical experiment, 100 cells were simulated in Matlab, neglecting movement, growth, division, and the spatial distribution of the cells. In both open and closed loop scenarios, the addition of a degradation tag reduces the dependence of X_T from the percentage of targets in the

consortium (see Figure S11a). In addition, as shown in Figure S11b, the addition of an enzymatic degradation can also reduce the average error at steady state of the multicellular architecture in both open and closed loop scenarios. As such, the addition of a degradation tag, although impairing the precision of the regulation in nominal condition, improves the robustness of the architecture with respect to changes in the consortium composition in closed loop. However, if the maximal rate of degradation is excessively high, the feedback is completely suppressed and the open and closed loop become equivalent.

S3.4. Effects of growth on the consortium dynamics

In the mathematical model presented in in section S3.1 we assumed the number of cells in the consortium to be fixed. This assumption, made to simplify the analytical study of the robustness of the architecture, was representative of a steady-state condition reached in batch cultures. However, growth can play a significant role in the dynamics of the consortium. As such, in this section we will investigate the effects that growth has on the overall dynamics and robustness of the architecture. This was done by complementing models (3.7) and (3.8) with the equations describing the growth of both populations. Assuming logistic growth due to limited availability of nutrients, the dynamics of N_t and N_c can be written as

$$\begin{aligned} \dot{N}_c &= k_c N_c \left(1 - \frac{N_c + N_t}{N_m}\right) \\ \dot{N}_t &= k_t N_t \left(1 - \frac{N_c + N_t}{N_m}\right), \end{aligned} \tag{3.24}$$

where k_c and k_t are the growth rates of the controller and target population, and N_m is the maximum density of the consortium.

The steady state solutions of equations (3.24) are the trivial equilibrium point (0,0), which is unstable, and a stable equilibrium set defined by the curve

$$N_c + N_t = N_m. \tag{3.25}$$

Even although the total density of the cells will reach N_m, the ratio of controllers to targets in the consortium depends on their initial concentrations.

We selected $k_t = 0.02$ so that the doubling time of the population is about 20 minutes (as done in [8]). Additionally, we empirically selected k_c =0.028 so that, based on the initial

target-to-controller ratio, the consortium's steady-state composition matches the data shown in Figure 4.

To evaluate how growth affects the consortium's dynamics and robustness, we extended the models from equations (3.7) and (3.8) by adding equations (3.24) to explicitly simulate growth in both the open and closed loop architectures. We then compared the time trajectories and steady states of $\tilde{X}_t^C$ and $\tilde{X}_t^O$ (defined in (3.21)) with the results from the original models that assume a constant number of cells. As shown in Figure S14, this comparison demonstrates that growth does not significantly affect the system's dynamics and performance in either configuration. To quantify the influence of growth on the consortium dynamics, we also computed the difference between the signals $\tilde{X}_t^C$ and $\tilde{X}_t^O$, which never exceeded 0.6542 for $\tilde{X}_t^C$ and 0.8493 for $\tilde{X}_t^O$.

S4. Orthogonality between *las* and *lux* systems

When choosing multiple quorum sensing pathway that need to operate concurrently, a crucial aspect needed for the correct operation of the system is orthogonality. In our architecture we chose the *las* system to implement the actuation signal responsible to activate the *gfp* production and the *lux* systems to implement the signaling pathway that feeds the state of the targets back to the controller population. The choice of these two systems was guided by the extension and compatibility of their operation ranges [9]. While the las system remains nearly entirely orthogonal to 3-O-C6, the lux system can be partially activated by 3-O-C12. Therefore, we examined whether the lux system could be effectively combined with 3-O-C12 in our design.

The expression of a gene x driven by a promoter whose activity is induced by a single quorum sensing molecule can be modelled as:

$$\dot{x} = \mu_0 + \frac{(\mu - \mu_0)\, Q^n}{K^n + Q^n} - \gamma x.$$

Here, Q is the input concentration of the quorum sensing molecule, n is the hill exponent, K is the dissociation constant, and γ is the dilution rate. In case there is an additional non-

orthogonal Quorum Sensing molecule, denoted here as Q_2, the dynamics of x can be modified as (see [10] for more information)

$$\dot{x} = \mu_0 + \frac{\mu\,(Q+\epsilon Q_2)^n}{K^n+(Q+\epsilon Q_2)^n} - \gamma x.$$

In this model ϵ measures the crosstalk strength. The functional relationship between the concentrations of Q and Q_2 and the steady state expression of x ($\bar{x}$) can be described as

$$\bar{x} = f(Q, Q_2) = \bar{\mu}_0 + \frac{\bar{\mu}(Q+\epsilon Q_2)^n}{K^n+(Q+\epsilon Q_2)^n}\ , \qquad (3.26)$$

where $\bar{\mu}_0 = \frac{\mu_0}{\gamma}$ and $\bar{\mu} = \frac{\mu-\mu_0}{\gamma}$.

To understand the influence of 3-0-C12 on the lux system, we parametrized the Hill activation function presented in equation (3.26) using the data available in [9]. Specifically, by posing $Q =$ [3-O-C6] and $Q_2 =$ [3-O-C12], we could directly take the values of $\bar{\mu}_0$, $\bar{\mu}$, n and K the Supplementary Table 4 in [9]. Additionally, defining $K_\epsilon = \frac{K}{\epsilon}$, ϵ can be computed as

$$\epsilon = \frac{K_\epsilon}{K}.$$

This value can be computed using the data present in the Supplementary Table 4 [9] recalling that K_ϵ is such that $f(0, K_\epsilon) = \bar{\mu}_0 + \frac{\bar{\mu}}{2}$.

Given the function $f(Q, Q_2)$ parametrized on the data available in [9], we computed

$$e = \frac{f(Q,Q_2)-f(Q,0)}{f(Q,0)},$$

which measures the difference in activation between the scenario without cross-talk and the one where cross-talk is explicitly modelled. Low values indicate that cross-talk has minimal impact on activation. We investigated the values of e for ([3-O-C6],[3-O-C12])∈ $[10^{-10}\ M, 10^{-8}\ M] \times [10^{-10}\ M, 10^{-8}\ M]$, which correspond to the working conditions for both molecules (see [9] and Figure 2). As shown in figure S15, the index is significantly greater than 0 only when [3-0-C12] >> [3-0-C6]. However, this condition is never reached in our architecture as to high levels of [3-O-C12] correspond high levels of [3-O-C6] (3-O-C12 induces *luxI* that produces 3-O-C6). This result demonstrates that the lux and las systems can be used together in our design, as they effectively function as orthogonal quorum sensing modules.

SUPPLEMENTARY TABLES

Primer Name	Primer sequence (5′- 3′)
**Sigma_plus_tag_F**	GAGGAGAAATACCATAATCC
**Sigma_plus_tag_R**	GTTTTATCAGACCGCTTC
**placUV5_F**	TGGTTTCACATTCACCAC
**placUV5_R**	GCTAGATCTAGAGAAATTGTTATCCGCTCACA
**Anti_Sigma_plus_tag_F**	ATGTCATATGCCGCGATAGAGGAGGTAA
**Anti_Sigma_plus_tag_R**	CTCTCATCCGCCAAAACA
**Back_extralac_for**	CACCCCAGGCTTTA
**Back_extralac_rev**	ATTCGGGATCGAGAT
**Ins_extralac_for**	GAATTCCGATTCATTAATG
**Ins_extralac_rev**	CGGAAGCATAAAGTGT
**RFP_Afe_for**	TGATCGAGCGCTGATAAGTCCCTAACTTTTACAGC
**RFP_Nde_rev**	TGCTCACATATGGGACCAAAACGAAAAAAGGC
**p_Las_Lux_GFP_3_0_fwd_20**	ACGCCCTTGCAGCGTAATAATACTAGAGAAAGAG GAGAAATACTAGATGCG
**p_Las_Lux_GFP_3_0_rev_20**	CTCTATCGCGGAAATTGACACTCGGCGTTATGTC ATGAAG
**luxI_for**	TGTCAATTTCCGCGATAGAGGAG
**luxI_rev**	TTATTACGCTGCAAGGGCGTA

Table S1 Name and sequence of the primers used for the cloning of the constructs used in this study. The postscripts R and rev identify the reverse primers while the suffixes F and for represent the forward primers. The cloning where each primer has been used is reported in the section *Strains and constructs* of the main text.

**Gene Name**	**Sequence (5′ – 3′)**
**RFP**	GATAAGTCCCTAACTTTTACAGCTAGCTCAGTCCAGGTATTATGC TAGCCTGAAGCTGTCACCGGATGTGCTTTCCGGTCTGATGAGTCC GTGAGGACGAAACAGCCTCTACAAATAATTTTGTTTAATACTAGA GAAAGAGGGGAAATACTAGATGGTTTCCAAGGGCGAGGAGGAT

	AACATGGCTATCATTAAAGAGTTCATGCGCTTCAAAGTTCACATG<br>GAGGGTTCTGTTAACGGTCACGAGTTCGAGATCGAAGGCGAAGG<br>CGAGGGCCGTCCGTATGAAGGCACCCAGACCGCCAAACTGAAA<br>GTGACTAAAGGCGGCCCGCTGCCTTTTGCGTGGGACATCCTGAGC<br>CCGCAATTTATGTACGGTTCTAAAGCGTATGTTAAACACCCAGCG<br>GATATCCCGGACTATCTGAAGCTGTCTTTTCCGGAAGGTTTCAAG<br>TGGGAACGCGTAATGAATTTTGAAGATGGTGGTGTCGTGACCGTC<br>ACTCAGGACTCCTCCCTGCAGGATGGCGAGTTCATCTATAAAGTT<br>AAACTGCGTGGTACTAATTTTCCATCTGATGGCCCGGTGATGCAG<br>AAAAAGACGATGGGTTGGGAGGCGTCTAGCGAACGCATGTATCC<br>GGAAGATGGTGCGCTGAAAGGCGAAATTAAACAGCGCCTGAAA<br>CTGAAAGATGGCGGCCATTATGACGCTGAAGTGAAAACCACGTA<br>CAAAGCCAAGAAACCTGTGCAGCTGCCTGGCGCGTACAATGTGA<br>ATATTAAACTGGACATCACCTCTCATAATGAAGATTATACGATCG<br>TAGAGCAATATGAGCGCGCGGAGGGTCGTCATTCTACCGGTGGC<br>ATGGATGAGCTGTACAAATAACTCGGTACCAAATTCCAGAAAAG<br>AGGCCTCCCGAAAGGGGGGCCTTTTTTCGT TTTGGTCC

Table S2 Names and sequences of the gene commercially synthesized for this study. The RFP sequence synthesized by Thermo Fisher scientific and used to embed a constitutively expressed red reporter in the controllers, as described in the section *Strains and constructs* of the main text .

IPTG CONCENTRATION	0 μM	3 μM	5 μM	7 μM
0 μM	-	0.219	1.000	0.973
3 μM	0.219	-	0.179	0.098
5 μM	1.000	0.179	-	0.965
7 μM	0.973	0.098	0.965	-

Table S3 p-values of a multiple comparison analysis run on the slope of the linear least square estimators fitted on closed loop data spanning different consortium compositions. Details on

how the data were obtained can be found in section Consortium composition assays of the Methods.

IPTG CONCENTRATION	0 μM	3 μM	5 μM	7 μM
0 μM	-	0.9955	0.2535	0.8145
3 μM	0.9955	-	0.3711	0.9133
5 μM	0.2535	0.3711	-	0.8045
7 μM	0.8145	0.9133	0.8045	-

Table S4 p-values of a multiple comparison analysis run on the slope of the linear least square estimators fitted on closed loop data spanning different consortium compositions (non-normalized data). Details on how the data were obtained can be found in section Consortium composition assays of the Methods.

SUPPLEMENTARY FIGURES

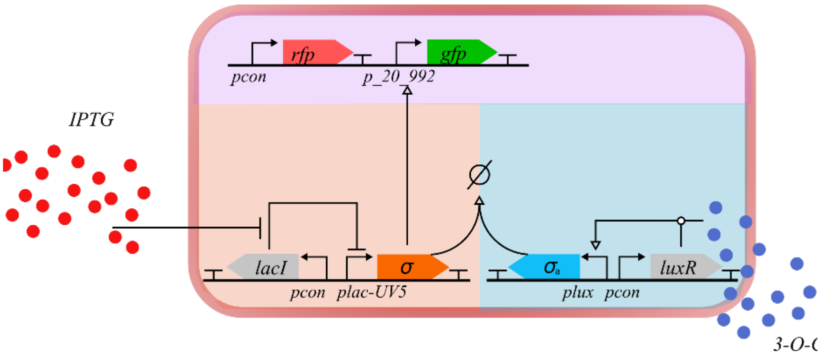

Figure S1 Schematic representation of the controllers used to characterise the input-output response. In this population the plasmid expressing lasI under the p_20_992 promoter was modified substituting lasI with a green fluorescent protein (GFP). The Synthetic Biology Open Language (SBOL) notation is used to denote promoters, genes, promotion/inhibition relationships and quorum sensing molecules. The shaded areas identify different functional modules within each population. Specifically, the purple area defines the module responsible for the generation of the output signal, the orange identifies the plasmid able to sense the concentration of the reference signal and the blue module is responsible for sensing the amount of 3-O-C6 HSL present in the growth media.

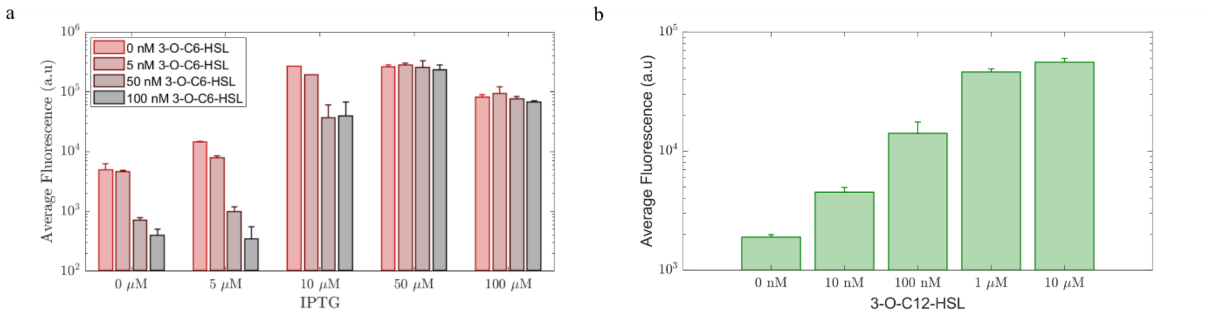

Figure S2 Steady state response of the original controllers and targets in single strain cultures. **a**. Average fluorescence of the controller population induced using different concentrations of 3-O-C6-HSL and IPTG. The data were collected 6h after the initial inoculation. The bar and vertical lines depict the average and standard deviation of the fluorescence expressed by the controllers over n=2 biological replicates. Different colors identify different levels of 3-O-C6 HSL supplemented to the growth medium. **b**. Average fluorescence of the targets induced using different concentrations of 3-O-C12-HSL. The data were collected 6h after the initial inoculation. The bar and vertical lines depict the average and standard deviation of the fluorescence expressed by the fluorescent controllers over n=2 biological replicates.

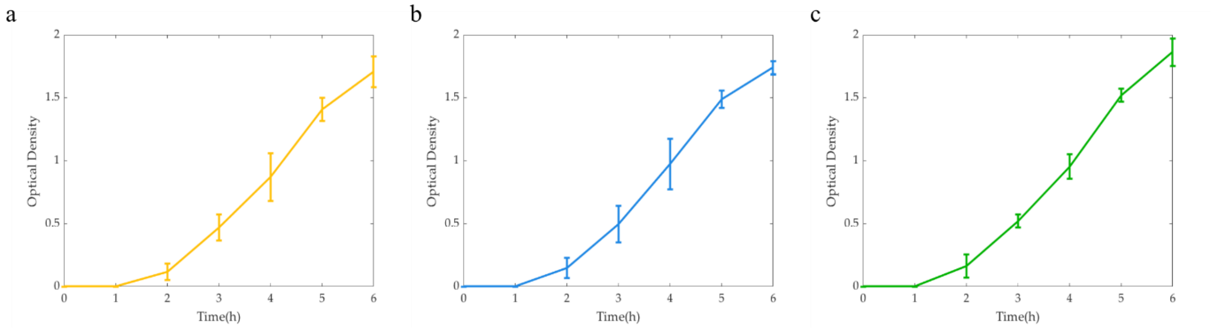

Figure S3 Growth curves of Targets (c), controllers (a) and open loop controllers (b) during the time course experiment reported in Figure S4. In each panel the solid lines and bars represent the average OD and the standard deviation at each time instant over n=3 biological replicates.

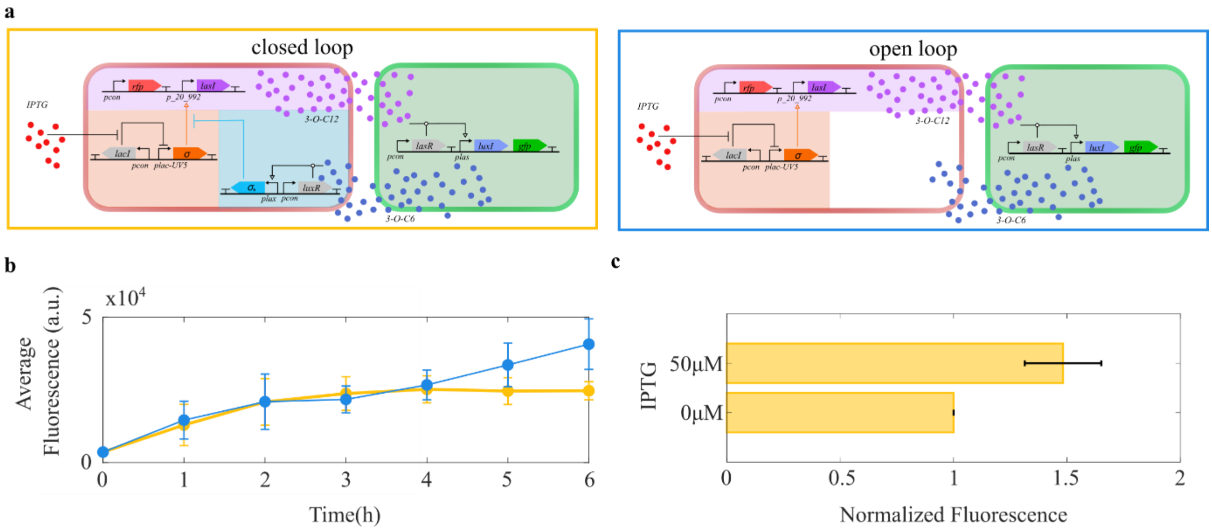

Figure S4 **a:** Schematic representation of the closed loop (left) and the open loop (right) configurations. **b:** Average fluorescence of the targets over a 6h time course in open (blue) and closed (yellow) loop using our original constructs. The solid dots and vertical bars represent the average and standard deviation over n=3 biological replicates, respectively. **c:** Normalized average fluorescence of the targets mixed with the controllers in closed loop when 0 μM and 50 μM IPTG were present in the growth media. The data were collected 6h after the initial inoculation. The fluorescence was normalized to the average fluorescence reached when no IPTG was added in the media. All data are averaged over n=3 biological replicates.

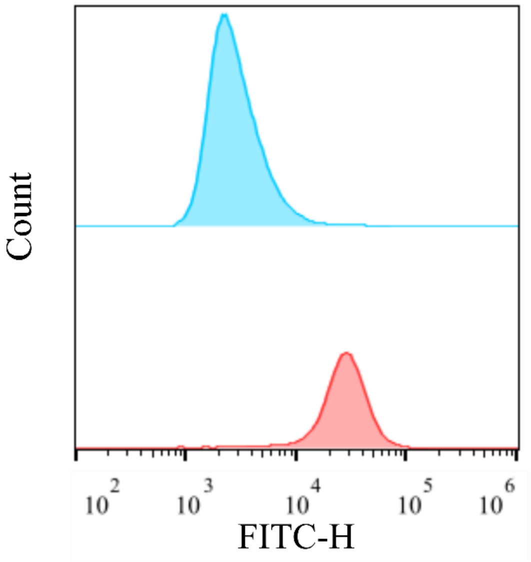

Figure S5 Fluorescence profile of the targets in monostrain culture (blue) and in co-culture with the original controllers (red) 6h after the initial inoculation. In both conditions no IPTG or 3-O-C12 were added to the growth medium. The data were collected using the protocol described in section *Sample preparation and analysis using flow cytometry* and analysed using FlowJo.

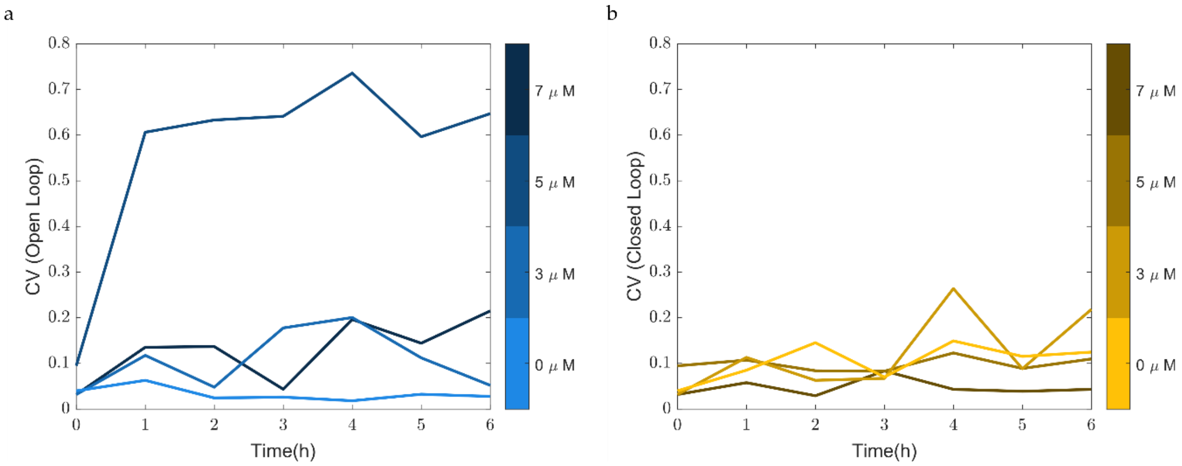

Figure S6 Comparison of the Coefficient of Variation (CV) during time course experiments in open loop (a) and closed loop configurations. The data are color coded according to the concentration of IPTG used during the experiment. The CV of experimental data was computed over n=3 biological replicates.

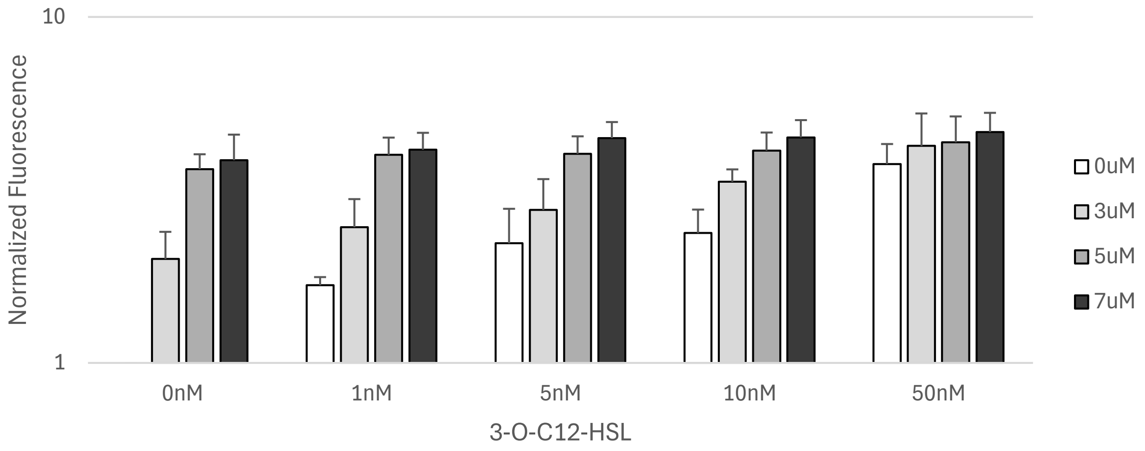

Figure S7 Normalized fluorescence levels of the target population when increasing levels of 3-O-C12 HSL were added to the culture media. The targets were mixed with the open loop controllers 2.0. and induced using different IPTG concentrations. The data were collected after a 6h incubation at 37 °C, shaking at 250rpm. All data were normalized to the 0nM 3OC12 + 0uM ITG condition. Different colors correspond to different IPTG concentrations supplemented to the growth medium

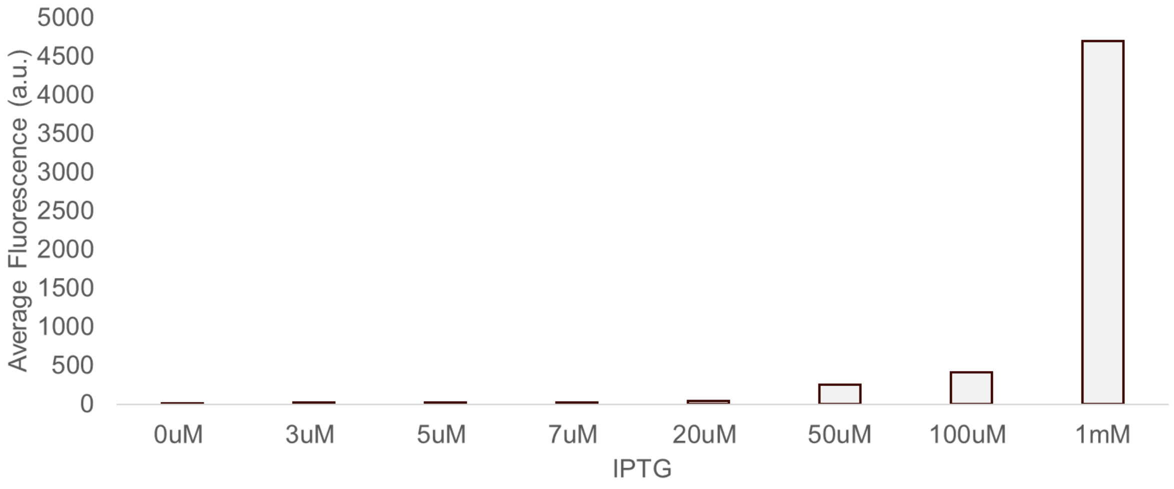

Figure S8 Average fluorescence levels of the controllers 2.0 population where *lasI* was substituted with a *gfp* expression gene. The data were collected after a 3h incubation at 37 °C, shaking at 250rpm in LB supplemented with the indicated concentrations of IPTG.

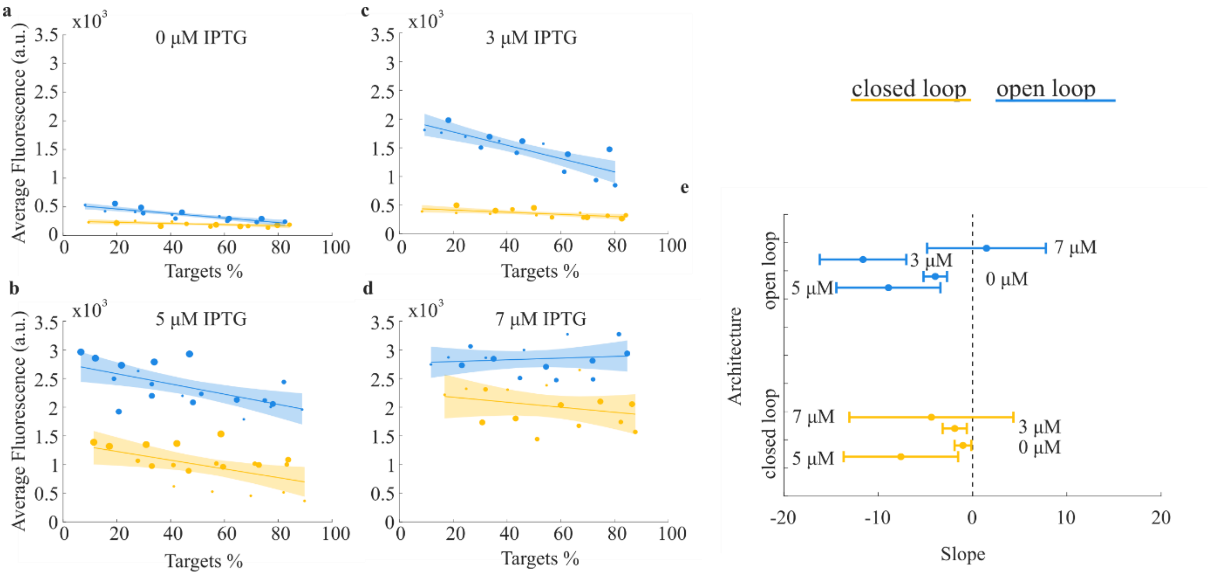

Figure S9 **a-d.** Average fluorescence in open (blue) and closed (yellow) loop across different consortium compositions using controllers 2.0. /open loop controllers 2.0. The solid dots are the data collected over n=3 biological replicates. Dots of the same size belong to the same replicate. The data were fitted with a first order polynomial. The fitting and its confidence intervals are represented with the solid lines and the shaded areas, respectively. **e.** Comparison of the estimates of the slopes of the linear fittings of data in panels **a-d** when a closed (yellow) or open (blue) loop architecture was used. The solid dots represent the slope estimates and the horizontal bars the 90% confidence interval on the estimates. We compared the slopes of the lines fitting the open and closed loop datapoints at different IPTG concentrations using analysis of covariance and a multiple comparison test. When 0µM and 3µM IPTG were used, the slopes of the linear models fitting the closed and open loop datapoints were statistically different with p-values equal to 0.00032 and 0.00014, respectively. In contrast, when 5µM and 7µM IPTG were present in the culture media, the changes were not significant. Using the same methodology we also compared the closed

loop slopes at different IPTG levels, finding no significant differences within the closed loop system (detailed p-values provided in Table S4).

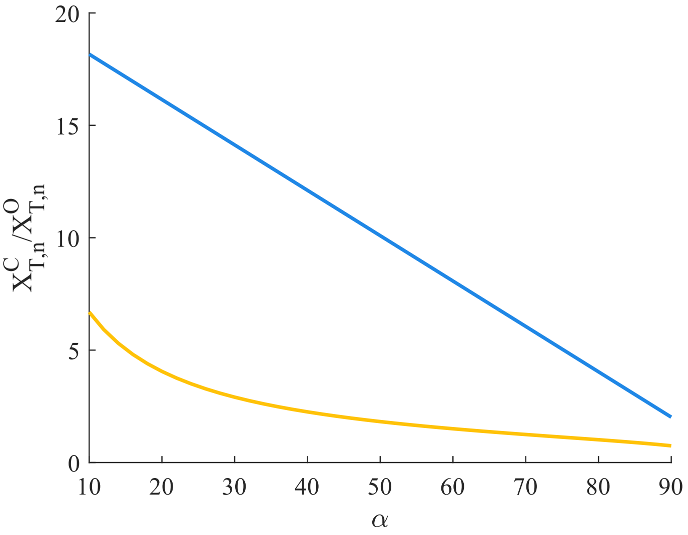

Figure S10 Dependence of steady state expression of $X^C_{T,n}$ (yellow) $X^O_{T,n}$ (blue) on the percentage of targets in the consortium α. The curves are obtained by simulating model (3.1) in open and closed loop using as values of the parameters $\mu = 400, r = 3, \gamma_z = 0.01, \theta = 0.3, \eta = 2, \gamma = 0.023, \beta_I = 0.0076, \beta_T = 0.03, \beta_X = 0.03$ (taken from [4]).

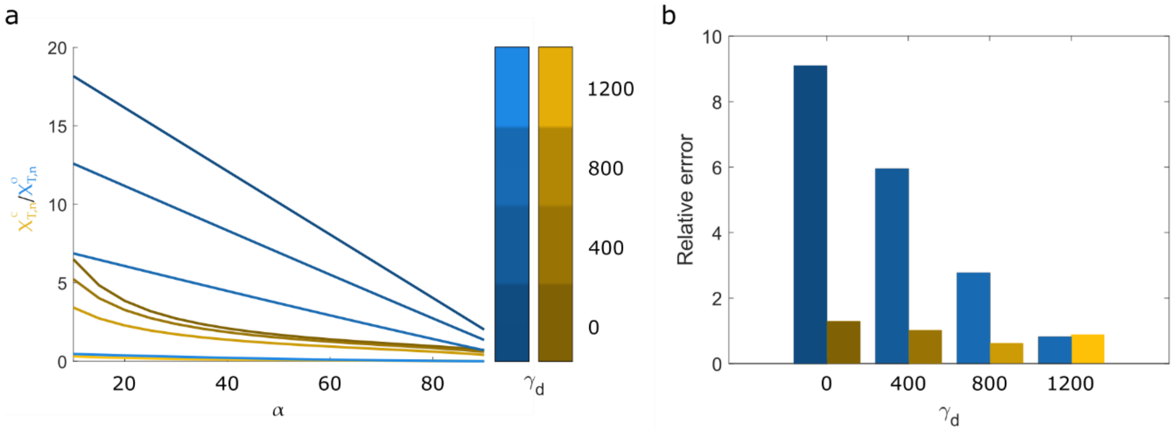

Figure S11 **a.** Dependence of steady state expression of $X^C_{T,n}$ (yellow) $X^O_{T,n}$ (blue) on the percentage of targets in the consortium α, varying the maximal enzymatic degradation rate

γ_d. The curves are obtained by simulating model (3.23) in open and closed loop using as values of the parameters $\mu = 400, r = 3, \gamma_z = 0.01, \theta = 0.3, \eta = 2, \gamma = 0.023, \beta_I = 0.0276, \beta_T = 0.03, \beta_X = 0.03,$ (taken from [4]), $K_d = 0.01$ (taken from [7]). **b.** Average relative steady state error $\left(\frac{X_{T,n}^{C/O} - \mu r/\theta}{\mu r/\theta}\right)$ over all consortium composition in open and closed loop for different values of the maximal enzymatic degradation rate γ_d.

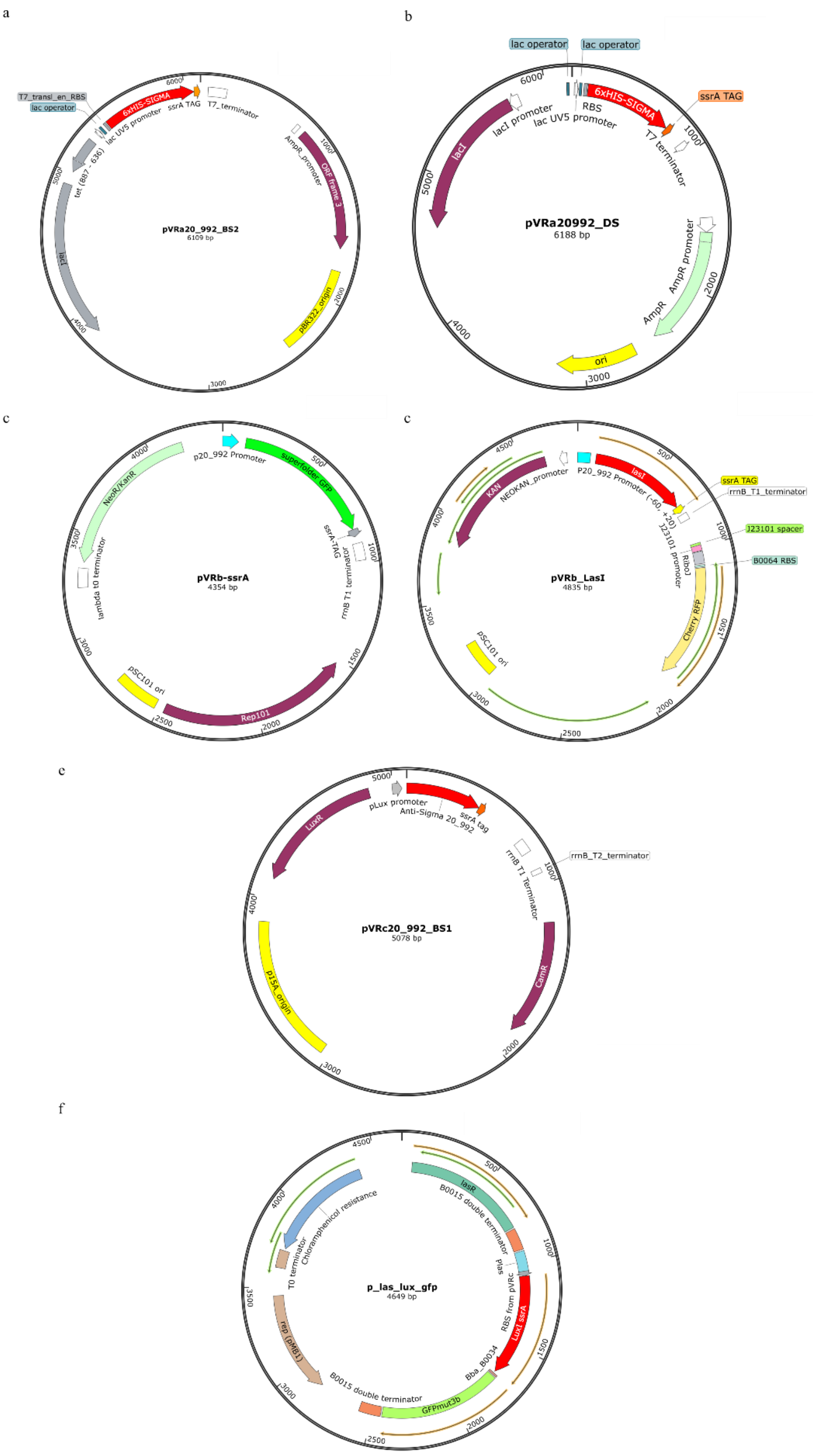

Figure S12 Plasmid maps of the construct used in this study. **a** map of the pVRa_20_992_BS2 (σ producing plasmid of the controllers) **b** map of the pVRa_20_992_DS (σ producing plasmid

of the controllers 2.0) **c** map of the pVRb_ssrA (plasmid producing GFP under the p_20_992 promoter) **d** map of the pVRb_lasI_RFP (plasmid producing LasI under the p_20_992 promoter) **e** map of the pVRc_20_992_BS1 (plasmid producing anti-σ) **f** map of the plas_lux_GFP_3.0 (plasmid embedded in the targets). The construction of these plasmids is reported in section *Strains and constructs* of the main text. The controllers are constructed transforming *E.coli* MG1655 with pVRa_20992_BS2, pVRb_lasI_RFP and pVRc_20992_BS1. The controllers 2.0. are constructed transforming *E.coli* MG1655 with pVRa_20992_DS, pVRb_lasI_RFP and pVRC_20992_BS1. The Targets are constructed transforming *E.coli* MG1655 with plas_lux_GFP_3.0.

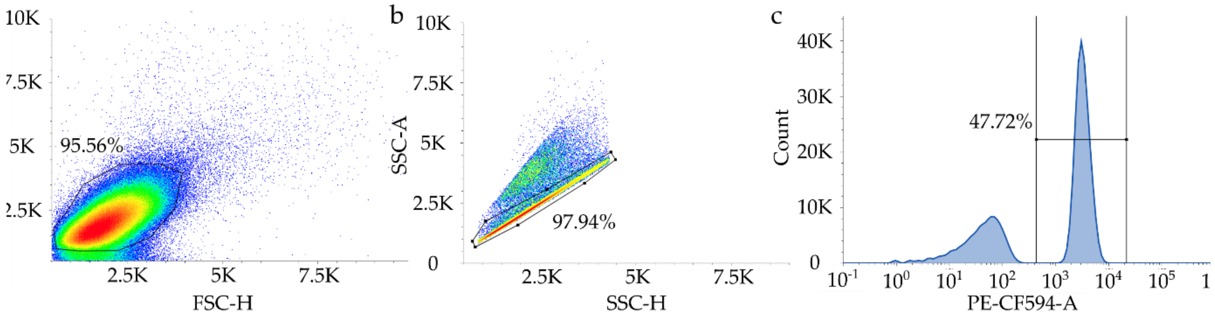

Figure S13 **a.** Density plot of events in the FSC-H,SSC-H plane. Red areas represent zones of the plane with more events. The Polygons drawn represent events classified as healthy cells (black). **b.** Density plot healthy cells in the SSC-H,SSC-A plane. Red areas represent zones of the plane with more events. The black polygon drawn is the gate used to select single cells against aggregates. **c.** Histogram of single cells in the PE-CF594-A (Red Fluorescence) channel. The horizontal gate highlights controllers in the consortium.

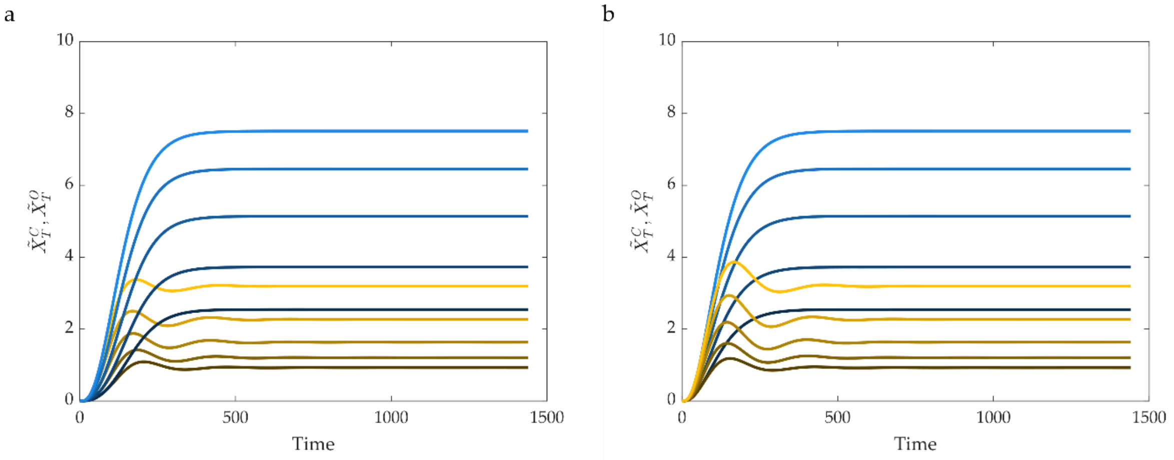

Figure S14 Comparison of $\tilde{X}_t^C$ (yellow) and $\tilde{X}_t^O$ (blue) between a model where growth is explicitly modelled (a) and a model where the number of cells is assumed to be constant (b). Each line correspond to different initial targets-to-controllers ratios in the consortium. **a.** The initial cell concentrations were set to achieve target-to-controller ratios of 15:1, 8:1, 4:1, 2:1, and 1:1, corresponding to the curves from darkest to lightest. k_t, k_c and N_m are selected as described in section S3.4. **b.** The initial concentration of cells in the consortium was set as the steady state concentration of targets and controllers in the simulation with the same colour code in panel **a**.

In both panels the parameters and initial conditions of the gene networks are selected as in Figure S10.

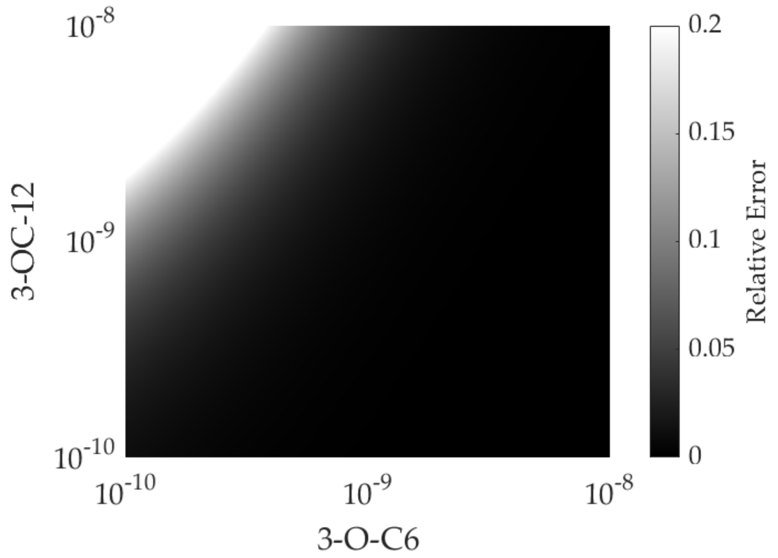

Figure S15 Relative error within the effective ranges of 3-O-C6 and 3-O-C12. The relative error is calculated by comparing induction level without crosstalk to those with crosstalk.

References

(1) Chevalier, M., Gómez-Schiavon, M., Ng, A. H., and El-Samad, H. (2019) Design and Analysis of a Proportional-Integral-Derivative Controller with Biological Molecules. *Cell Systems 9*, 338-353.e10.
(2) Briat, C., Gupta, A., and Khammash, M. (2016) Antithetic Integral Feedback Ensures Robust Perfect Adaptation in Noisy Biomolecular Networks. *Cell Systems 2*, 15–26.
(3) Qian, Y., and Vecchio, D. D. Realizing 'integral control' in living cells: how to overcome leaky integration due to dilution?
(4) Martinelli, V., Salzano, D., Fiore, D., and Di Bernardo, M. (2022) Multicellular PI Control for Gene Regulation in Microbial Consortia. *IEEE Control Syst. Lett. 6*, 3373–3378.
(5) Aoki, S. K., Lillacci, G., Gupta, A., Baumschlager, A., Schweingruber, D., and Khammash, M. (2019) A universal biomolecular integral feedback controller for robust perfect adaptation. *Nature 570*, 533–537.
(6) Zand, A. M., Tavazoei, M. S., and Kuznetsov, N. V. (2022) Chaos and Its Degradation-Promoting-Based Control in an Antithetic Integral Feedback Circuit. *IEEE Control Syst. Lett. 6*, 1622–1627.
(7) Annunziata, F., Matyjaszkiewicz, A., Fiore, G., Grierson, C. S., Marucci, L., Di Bernardo, M., and Savery, N. J. (2017) An Orthogonal Multi-input Integration System to Control Gene Expression in *Escherichia coli*. *ACS Synth. Biol. 6*, 1816–1824.
(8) Matyjaszkiewicz, A., Fiore, G., Annunziata, F., Grierson, C. S., Savery, N. J., Marucci, L., and Di Bernardo, M. (2017) BSim 2.0: An Advanced Agent-Based Cell Simulator. *ACS Synth. Biol. 6*, 1969–1972.
(9) Kylilis, N., Tuza, Z. A., Stan, G.-B., and Polizzi, K. M. (2018) Tools for engineering coordinated system behaviour in synthetic microbial consortia. *Nat Commun 9*, 2677.
(10) Scott, S. R., Din, M. O., Bittihn, P., Xiong, L., Tsimring, L. S., and Hasty, J. (2017) A stabilized microbial ecosystem of self-limiting bacteria using synthetic quorum-regulated lysis. *Nat Microbiol 2*, 17083.
